# Results and lessons learnt from a randomized controlled trial: prophylactic treatment of vestibular migraine with metoprolol (PROVEMIG)

**DOI:** 10.1186/s13063-019-3903-5

**Published:** 2019-12-30

**Authors:** Otmar Bayer, Christine Adrion, Amani Al Tawil, Ulrich Mansmann, Michael Strupp, PROVEMIG investigators, Michael Strupp, Hans-Christoph Diener, Hubert Löwenheim, Thomas Lempert, Wolfgang Heide, Holger Rambold

**Affiliations:** 1 German Center for Vertigo and Balance Disorders (DSGZ), Ludwig Maximilians University, Campus Grosshadern, Munich, Germany; 2 ReliaTec GmbH, Garching, Germany; 3 Institute for Medical Information Processing, Biometry and Epidemiology (IBE), Ludwig Maximilians University, Campus Grosshadern, Marchioninistr. 15, 81377 Munich, Germany; 4 Department of Neurology, Ludwig Maximilians University, University Hospital Munich, Campus Grosshadern, Munich, Germany

**Keywords:** Vestibular migraine, Episodic migraine, Patient-centred outcomes, Symptom diary, Pharmaceutical intervention, Pharmacological prophylaxis, Randomized controlled trial, Comparative effectiveness

## Abstract

**Background:**

Vestibular migraine (VM) is the most frequent cause of recurrent spontaneous attacks of vertigo causally related to migraine. The objective of the Prophylactic treatment of vestibular migraine with metoprolol (PROVEMIG) trial was to demonstrate that metoprolol succinate is superior to placebo in the prevention of episodic vertigo- and migraine-related symptoms in patients with VM.

**Methods:**

This phase III, two-arm, parallel-group, double-blind, randomized placebo-controlled trial was designed to be conducted at tertiary referral centres at neurology and ear, nose and throat departments of eight German university hospitals. The planned sample size was a total of 266 patients to be allocated. Adults aged 18 years or above diagnosed with probable or definitive VM according to the Neuhauser criteria 2001 were randomly assigned 1:1 to 6 months blinded metoprolol (maintenance dosage of 95 mg daily) or placebo. The primary efficacy outcome was the self-reported number of vertiginous attacks per 30 days documented by means of a paper-based daily symptom diary. The pre-specified time period of primary interest was defined as months 4 to 6. Secondary outcomes included the patient-reported number of migraine days and vertigo days, the Dizziness Handicap Inventory, and clinical assessments. Adverse events were reported throughout the whole 9-month study period.

**Results:**

At the time of trial termination, no evidence for a difference in the incidence of vertiginous attacks between groups was detected. For the full analysis set, the incidence rate ratio was 0.983 (95% confidence interval (CI) 0.902–1.071) for metoprolol versus placebo. In both groups, there was a significant decline over time in the overall monthly vertigo attacks by a factor of 0.830 (95% CI 0.776–0.887). Results were consistent for all subjective and objective key measures of efficacy. The treatment was well tolerated with no unexpected safety findings.

**Conclusions:**

After randomizing 130 patients PROVEMIG had to be discontinued because of poor participant accrual not related to the tolerability of the study medication or safety concerns; no treatment benefit of metoprolol over placebo could be established. Additional preparatory work is much needed in the development, psychometric evaluation and interpretation of clinically meaningful end points in trials on episodic syndromes like VM taking into consideration the complexity of this disease entity comprising two domains (vertigo- and headache-related disability).

**Trial registration:**

EudraCT, 2009-013701-34. Prospectively registered on 8 April 2011.

**Table:** 

**Lessons learnt**	
▪ Thus far, there are no definitive specific curative or preventative therapies available for vestibular migraine (VM). ▪ This is the first pragmatic phase III, double-blind, randomized placebo-controlled superiority trial in adults with definite or probable VM comparing metoprolol as a prophylactic medication against placebo. ▪ There are important implications for the planning stage of future randomized controlled trials in VM with respect to placebo or nonspecific and drug-specific effects. ▪ The PROVEMIG trial was prematurely ended due to insufficient recruitment. Reasons for the poor participant accrual were multifactorial and included lowered patient acceptability, unwillingness to accept the underlying intervention (being on antihypertensive therapy), comorbidities being contraindications for metoprolol, or uncertainties in diagnosis. ▪ For VM, there is a strong need to develop and validate clinically meaningful, consensus-based patient-centred core outcome measures considering both the vestibular- and headache-related disease burden and to assess their psychometric performance.	

## Background

During the past decades, vestibular migraine (VM) has been identified as a type of migraine with the leading symptom of vertigo. Recently, it has been accepted as a distinct diagnostic entity by the Bárány Society and the International Headache Society [1]. Since vertigo frequently occurs in isolation and is not always accompanied by a headache or other migrainous symptoms, there is a strong need for accepted diagnostic criteria; these were first published in 2001 by Neuhauser and colleagues [2] and later refined by the International Classification of Vestibular Disorders of the Bárány Society [3].

Based on validated neuro-otologic interviews [4, 5], the prevalence of migrainous vertigo in the general adult population was estimated in a large German neuro-otologic survey; its lifetime prevalence was 0.98% and the 12-month prevalence 0.89% [6]. The similarity of these two numbers suggests that these patients suffer chronically from this condition. A more recent survey from the USA found a prevalence of 2.7% in adults [7]. In a specialized dizziness clinic, VM is the most frequent cause of spontaneous recurrent attacks of vertigo and accounts for approximately 10% of the patients [8]. The majority of patients with VM are middle-aged and in the middle of their working lives.

With a lack of double-blind, randomized controlled clinical trials [9], recommendations for treatment are aligned to those of migraine [10, 11]. The following drugs have been recommended as prophylactic treatment for VM: beta blockers, valproic acid, lamotrigine [12], tricyclic antidepressants and topiramate [13]. In an observational study on 81 patients, the effects of tricyclic antidepressants, beta blockers, or calcium-channel blockers in combination with diet were evaluated. Seventy-two percent of the patients showed a good response [14]. More recently, flunarizine [15], propranolol and venlafaxine [16] have been investigated in active-controlled, open-label trials. Metoprolol is listed as a group 1 medication (drug of first choice) in a dose of 50 to 200 mg and has shown efficacy in prophylactic treatment of migraine [17, 18]. Due to the absence of consensus guidelines for the treatment of VM, beta blockers such as metoprolol are commonly prescribed as off-label preventive pharmacologic treatment in VM.

For this reason, the Prophylactic treatment of vestibular migraine with metoprolol (PROVEMIG) trial was conducted. This investigator-initiated, prospective, longitudinal, national, multicentre, double-blind, randomised, placebo-controlled, two-arm parallel group, phase III, pragmatic superiority trial aimed to evaluate the effectiveness, safety and tolerability of metoprolol succinate versus placebo for the preventive treatment of VM. Treatment duration in both arms was 6 months, with a 3-month post-treatment follow-up period. The primary objective was to demonstrate the superiority of metoprolol with respect to the incidence rate of vertiginous attacks. Further secondary objectives included comparing both regimens with respect to vertigo and headache characteristics, to investigate changes in neurological and neuro-ophthalmological assessments, and vertigo-related impairment of quality of life, and to further establish the safety profile of the drug. We report the pre-specified efficacy and safety analyses for the 6-month treatment period following reporting guidelines for trials describing patient-reported outcomes and related extensions (the checklist for the CONSORT 2010 statement is provided as Additional file 1) [19–21].

## Methods

### Study oversight

The study was investigator-initiated and conducted in accordance with the principles of the Declaration of Helsinki, the International Conference for Harmonisation Guidelines for Good Clinical Practice, and relevant national regulations. The protocol (Additional file 2) was approved by the ethics committees of each participating centre. Furthermore, the efficacy, safety, integrity and feasibility of the trial were monitored by a Data Safety Monitoring Board consisting of three independent, non-participating clinicians. All patients provided written informed consent before any study procedures or assessments were performed.

### Study population and procedures

Subjects were screened at six German academic outpatient clinics; four of these investigational sites (German Center for Vertigo and Balance Disorders at the University Hospital Munich; Department of Neurology of the General Hospital Celle; the University of Essen; and the community hospital at Altötting-Burghausen) allocated patients between 20 June 2012 (first patient) and 10 April 2017; the last patient visit was on 3 January 2018.

The patient population consisted of male or female patients aged 18 years and above diagnosed as having probable or definite VM according to the Neuhauser criteria [2] (see Additional file 2 for details). For enrolment, patients had to experience a frequency of 6 to 30 VM-related attacks per 3 subsequent months prior to the screening visit (information retrospectively collected at in-person interviews), be capable of following the study instructions and be likely to complete the study visits.

Exclusion criteria were a diagnosis of other co-existing vestibular disorders such as Menière’s disease, phobic postural vertigo, benign paroxysmal positional vertigo (BPPV) and vestibular paroxysmia. Patients were also excluded if they had central disorders such as paroxysmal brainstem attacks and transient ischemic attacks. Other exclusion criteria were contraindications for the treatment with metoprolol such as a known allergic reaction to the trial drug or other beta-receptor blockers, shock, acidosis, any bronchospastic disease (e.g. bronchial asthma), sick sinus syndrome, known sino–atrial or atrio–ventricular block, bradycardia of less than 50 bpm at rest, systolic blood pressure less than 100 mmHg, end-grade peripheral arterial disease, known severe coronary heart disease or heart failure and concurrent treatment with monoamine oxidase inhibitors, sympathomimetic drugs, catecholamine-depleting drugs, or digitalis glycosides. Other patient factors leading to exclusion included poorly controlled diabetes mellitus, pheochromocytoma, suspicion of developing thyrotoxicosis, disorders of homeostasis, porphyria, psoriasis, pregnancy or breastfeeding, persistent hypertension with systolic blood pressure higher than 180 mmHg or diastolic blood pressure higher than 110 mmHg (mean of three consecutive arm-cuff readings over 20 to 30 min) that cannot be controlled by antihypertensive therapy, life expectancy of less than 12 months, other serious illness that may confound treatment assessment, currently receiving beta blockers, enrolment in another clinical trial, and exposure to any investigational medication within 30 days prior to the baseline visit.

#### Study procedures

This study consisted of a screening visit, a 6-month treatment period, and a final evaluation at month 9 after a 3-month post-treatment follow-up period. Both the study examinations and treatment were performed in an outpatient setting. Based on the information from the screening visit, patients were either randomized or were excluded if they did not meet eligibility criteria. On the day of inclusion, patients received their study medication together with a paper-based diary to document VM-related symptoms on a daily basis over the 9-month observation period. Patients were seen at five scheduled clinic visits for protocol-specified evaluations at screening, baseline (day of inclusion), and at months 1, 3, and 6 (end of treatment period); three standardized telephone interviews were performed after 2, 4, and 5 months post-randomization for compliance checks with respect to treatment and diary documentation, and safety assessment.

All enrolled patients underwent a physical examination at the baseline visit, and at every clinic visit post-randomization non-invasive neurological and neuro-otological and neuro-ophthalmological examinations such as video-oculography including bithermal caloric testing, assessment of pursuit eye movement, gaze-holding, saccades and subjective visual vertical (SVV) were performed. For more details refer to the original protocol in Additional files 2 and 4. Furthermore, participants had to complete the paper-based, self-administered Dizziness Handicap Inventory (DHI), a 25-item validated questionnaire with a 3-point response scale to rate the self-perceived impact of dizziness on health-related quality of life [22, 23]. Possible DHI total scores range from 0 to 100 points, with higher values reflecting greater impairment. Results from the trial assessments were recorded in paper-based case report forms filled in by the study personnel at each clinical site.

#### Event-driven diary documentation

Dizziness event data were captured by means of the patient’s diary with entries made whenever symptoms associated with migrainous vertigo occurred. Patients were instructed to document the following items: time of onset, duration, severity and type of the vertigo symptom (rotatory or postural vertigo, gait unsteadiness, or light-headedness); occurrence of accompanying symptoms (headache, nausea, vomiting, photo- or phonophobia, diplopia, other visual symptoms, tendency to fall); any action taken including any medication use. A diary template (original German version together with an English translation) is provided in Additional file 3.

### Randomization, concealment and blinding

**Fig. 1:**
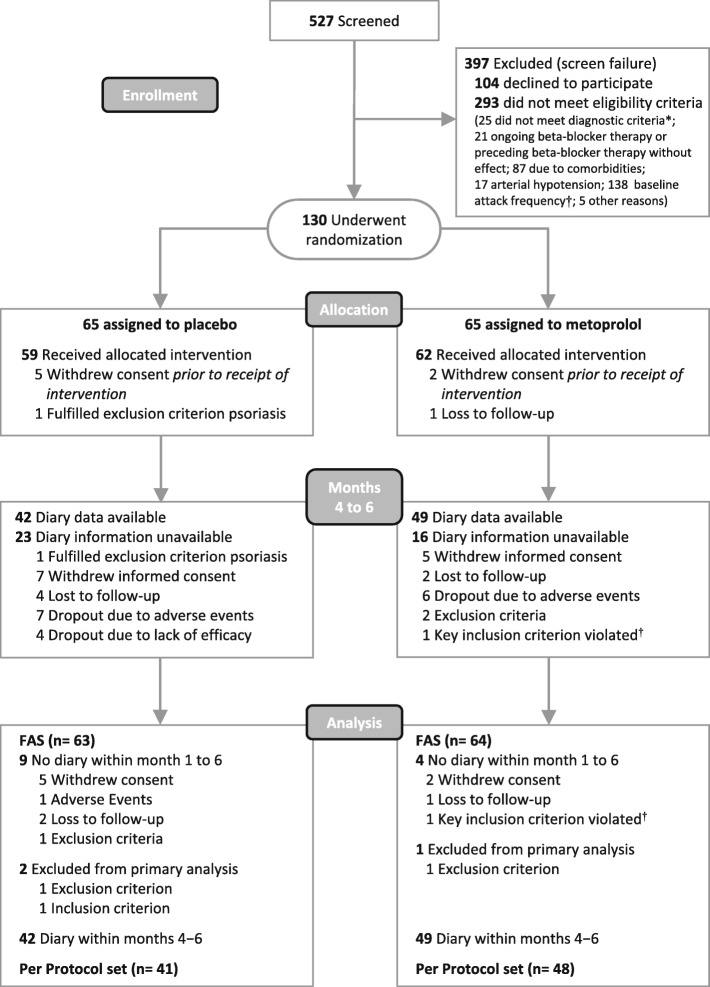
Consolidated Standards of Reporting Trials (CONSORT) 2010 flow diagram together with patient-reported outcome specific information. Enrolment and primary efficacy end points based on patient diaries. The steps lead from pre-screening to collection of the data used in the efficacy analyses. The diagram shows the extent of exclusions, loss to follow-up and missing data within the 6-month treatment period (diary information unavailable means no diary at all within the time period of primary interest, day 91 to day 180). *Diagnostic criteria according to Neuhauser 2001 [2]. ^†^Baseline frequency of vertigo attacks in the last 3 subsequent months prior to enrolment. Per protocol: treatment duration >90 days, counting from day of first intake. FAS full analysis set

Patients who met the eligibility criteria for enrolment were randomized in a 1:1 ratio to receive either metoprolol succinate or placebo for 6 months (Fig. 1). Each study site received a pool of study medication including the treatment assignment in an opaque, sealed emergency envelope. If an eligible patient dropped out before the study medication had been delivered they were replaced by the next eligible patient enrolled at the same site. The concealed allocation was performed by an internet-based randomization schedule stratified by study site (https://wwwapp.ibe.med.uni-muenchen.de/randoulette). The fixed block size was four (starting with 6) which was not disclosed during the trial. The random number list was generated by a person with no clinical involvement in the trial. Patients and site personnel including outcome assessors, data analysts and statisticians remained blinded to treatment allocation.

### Study treatments

Metoprolol succinate sustained-release tablets (Beloc-Zok® mite 47.5 mg; manufactured by AstraZeneca, Wedel, Germany) were encapsulated for blinding purposes. Hard gelatine capsules containing the active ingredient were refilled from original pharmacy packaging into re-labelled blisters by the pharmacy of the University Hospital in Heidelberg, Germany. Placebo was an identically appearing inactive capsule filled with mannitol and aerosil that did not contain any active ingredient; this was packed in blisters that looked identical to those of the investigational drug. Randomized patients were instructed to take one capsule per day starting as soon as possible after the receipt of the trial medication kit dispensed at the baseline visit. The treatment procedure included a 1-week run-in period of 47.5 mg metoprolol succinate or placebo once a day (up-titration), a 6-month maintenance dosage with 95 mg metoprolol succinate or placebo once a day, plus tapering with 47.5 mg metoprolol succinate or placebo once a day for 2 weeks before stopping the prophylactic therapy (down-titration). Placebo treatment was justified due to a lack of well-designed placebo-controlled trials for any drug therapy in VM. The 6-month treatment duration was deemed necessary to reliably assess a long-term prophylactic effect of the drug treatment on the incidence of VM-related vertigo attacks. If the patient was on prophylactic drug treatment for migraine, a washout period of at least 1 month was required before enrolment. Topiramate, valproic acid, lamotrigine, tricyclic antidepressants and other beta blockers were considered as prohibited concomitant medication and thus a protocol violation. Acute medical treatment of VM-related attacks such as episodic migraine with aura using non-opioid analgesics, non-steroidal anti-inflammatory drugs or triptans was allowed, serving as added rescue medication. We aimed to assess the comparative effectiveness of the assigned prophylactic treatment regardless of whether or not switching to rescue medication had occurred which can be denoted as ‘treatment policy estimand’ according to the International Council for Harmonisation E9 addendum [24].

### Statistical methodology and planned analyses

#### Protocol-defined efficacy outcomes and changes after trial commencement

The primary objective was to assess whether metoprolol was superior to placebo with respect to both disease domains of ‘vertigo’ and ‘headache’. For the purpose of the study, the target estimate was based on the overall monthly mean incidence of vertigo and headache attacks during a 3-month-long assessment period at the end of the double-blind, 6-month treatment period, i.e. months 4 to 6 (day 91 to day 180) was defined as the time period of primary interest assuming that the maximum treatment effect emerges after being on study medication for more than 3 months. The pre-specified primary efficacy outcomes were the patient-reported number of vertigo attacks and the number of headache attacks per 30-day interval (starting from time point 1 defined as the date of first intake of the study medication). According to the protocol, superiority was to be claimed based on the vertigo outcome domain alone. Thus, the incidence of headache attacks per 30 days was defined as a co-primary outcome. In case of claimed superiority with respect to the outcome domain ‘vertigo’, the comparison of the monthly incidence of headache attacks between both groups was to be considered next important.

However, due to the poor documentation concerning headache-related symptoms and the diary focussing on the vestibular symptoms, derivation of a measurable variable for headache attacks was considered impossible and the co-primary efficacy end point had to be omitted. Furthermore, the initially planned secondary outcomes duration and severity of vertigo episodes were omitted due to insufficient data quality. Instead, the number of vertigo days per 30 days (which was not pre-registered in the protocol) was defined as a clinically meaningful key secondary efficacy outcome to assess the disease burden with respect to the outcome domain ‘vertigo’. A vertigo day was defined as a calendar day (00:00 to 23:59) demonstrating at least one documented vertigo episode of at least 5 min (regardless of severity and type). Derivation of this efficacy variable relies on fewer assumptions compared to a vertigo attack and also enables handling missing diary items.

On the contrary, a vertigo attack was endorsed applying the following decision rules: duration of at least 5 min and no longer than 72 h, irrespective of vertigo type and severity; if time data (start and stop time) for a vestibular symptom were absent a duration of 24 h was assumed; a vertigo episode which was interrupted by sleep or temporarily remits was classified as one single attack, and not two; if applicable, patient-reported vertigo symptoms reported on 2 or more consecutive calendar days were summarized to one single vertigo episode lasting over consecutive calendar days (however, if the resulting duration extends beyond 72 h these calendar days were considered free of vertigo attacks but counted as vertigo days).

A pre-specified diary-based secondary efficacy outcome was the number of monthly headache days (a calendar day where headache of any severity occurred according to the patient ratings). During the blind data review, this patient-reported outcome was refined to as a migraine headache day (MHD), requiring at least one additional migraine-associated symptom such as nausea, vomiting, phono- or photophobia, disturbance of vision, or “migraine” provided as free text on the diary. However, features such as duration and severity of migraine headache or criteria as proposed by the International Classification of Headache Disorders-3 from 2018 [1] were not considered in order to derive definite MHD since these items were not requested on the diary.

All these changes to the latest protocol version concerning efficacy evaluation were made before breaking the treatment blind thus minimizing outcome reporting bias. Owing to the complexity of vestibular and migraine-associated symptoms, inaccurately documented episodes of vertigo (e.g. missing outcome items), and different individual perceptibility of both domains of the disease, the assessment of vertigo attacks, days and MHDs based on the raw daily diary recordings was very challenging. Therefore, a computer algorithm programmed in SAS was developed for the process of outcome adjudication and to derive all diary-based efficacy variables.

Protocol-defined observer-reported secondary efficacy end points included the proportion of patients achieving an improvement from baseline to month 6 in pursuit eye movement (change from ‘saccadic (of any direction)’ to ‘smooth’ versus change from ‘smooth’ to ‘saccadic’, or no change), and the proportion of patients achieving an improvement in SVV (change from ‘abnormal’ to ‘normal’ versus change from ‘normal’ to ‘abnormal’ or no change; ‘abnormal’ was defined as the absolute deviation of more than 2.5° from vertical). Furthermore, the absolute change in the DHI mean total score from baseline to month 6 was assessed.

#### Statistical efficacy analyses (including changes to protocol-specified analyses)

Efficacy analyses were conducted for the full analysis set (FAS) which included all randomized patients (intention-to-treat population) who did not fail to satisfy a major entry criterion, irrespective of whether they were treated or not. Subjects who provided neither primary nor secondary efficacy data were excluded from efficacy analyses, assumed missing at random (MAR). The per-protocol sample consisted of all participants who were part of the FAS who did not substantially deviate from the protocol and who were on treatment for more than 90 days, counting from the day of first intake. Safety analyses were performed on all patients who received at least one dose of study drug.

According to the protocol, the principal analysis for the primary end point incidence of vertigo attack per 30 days during the 3-month time period of primary interest (months 4 to 6) was a robust non-parametric comparison between both treatment groups by means of the Wilcoxon rank-sum test. However, in the course of the trial it was evident that drop-outs and incomplete diary documentation creating missing data could not be adequately handled by the intended test-based approach. In order to deal with the missing data structure over time we used a Poisson mixed effects model (Poi generalized linear mixed model) which not only yields unbiased parameter estimates when missing observations are MAR, but also provides reasonably stable results even when the assumption of MAR is violated [25–27].

For this longitudinal model-based approach, the log-transformed number of evaluated days per 30 days (defined as the number of calendar days with assessments recorded in the diary within a 30-day interval) was considered as an offset term in order to reflect missing diary information (e.g. for withdrawals) and to standardize the monthly incidence of vertigo attacks to 30 days for that month. Time (range 1 to 6) and treatment-by-linear-time interaction were used as fixed effects, together with patient-specific intercepts and slopes for time as normally distributed random effects. Assuming no rapid onset of effect staying stable over time, the main fixed effect for the treatment group was omitted. The target estimates consist of the decay rate for the placebo group (fixed effect for time) as well as the incidence rate ratios (IRRs) for the metoprolol group (treatment-by-time interaction) to assess if the magnitude of the difference between treatment groups varies over time. The latter can be interpreted as ‘speed of efficacy’ [28]; that is, whether the active agent may be distinguished from placebo by how quickly a reduction in the incidence of attacks was achieved. The same longitudinal model approach was applied for vertigo days serving as a supplementary analysis.

MHDs per 30 days were analysed with a negative binomial model (with an offset term for the corresponding number of evaluated days during the 90-day assessment period) using self-reported symptoms documented within months 4 to 6 only. An analysis of covariance for absolute change from baseline in DHI mean total score at month 6 was performed, which used a factor for treatment group and the baseline value as a covariate.

For the binary response measures of change in state from baseline in SVV and pursuit eye movement at month 6, a logistic regression analysis was conducted (1, from abnormal to normal; 0, otherwise).

#### Safety and tolerability

Adverse events (AEs) and tolerability were systematically assessed by evaluating reported AEs, physical examinations and concomitant medication use. The safety population included data from all randomized patients who received at least one dose of the investigational medicinal product during the double-blind treatment phase. Serious AEs (SAEs) were coded and summarized by the Medical Dictionary for Regulatory Activities (MedDRA) system organ class and preferred term. For some AEs, the exact starting date (day and/or month) was partially or completely missing. In order to deal with the different input accuracy or partial date issues, AEs were classified with respect to their occurrence (AEs emerging while on treatment versus post-treatment AEs) assuming the AE was experienced at the earliest possible date.

#### Sample size considerations

A fixed sample size calculation was performed for the primary efficacy outcome (number of vertigo attacks). A sample size of 106 patients in each group will have 80% power to detect a probability of 0.389 that an observation X_M_ is less than an observation X_P_ using a Mann–Whitney test with a 5% two-sided significance level. The probability of *P* (X_M_ < X_P_) = 0.611 was calculated with a presumed normal distribution and difference in means of 1 and a standard deviation of 2.5 (nQuery Advisor 7.0). On the basis of our experience with patient compliance in previous studies and routine treatment, we assumed a drop-out rate of about 20%. Thus, the fixed target sample size was a total of 266 patients (133 in each treatment group) to be allocated. Further detailed descriptions on how the sample size was calculated are provided in Additional file 2.

The study database was stored in SAS (Unix Version 9.2, SAS Institute, Cary, NC, USA). Statistical analyses were performed using the statistical software package R version 3.5.1 [29]. For the efficacy analyses we used the R package lme4 (version lme4_1.1–18-1) to fit frequentist generalized linear mixed effects models [30, 31]. All statistical tests were two-sided, with a significance level of 0.05.

## Results

### Premature termination of the trial

In June 2017, the sponsor-delegated person together with the responsible biometrician and the Data Safety Monitoring Board prompted an early termination of the study on the grounds of poor patient accrual after randomization of 130 patients, and not for any reasons related to safety.

Financial resources for the continuation of the PROVEMIG trial were no longer available due to lack of funding. To reach the a priori determined target sample size of 266 patients in total, further years and more recruiting sites would have been required, which was considered not feasible. Further concerns were the fact that the monthly recruitment rates in the participating sites were lower than anticipated and decreasing over time (Additional file 4: Figure S1). Overall, early stopping of the trial for feasibility reasons at the risk of generating an underpowered trial providing inconclusive data seemed justified.

### Patient disposition and baseline characteristics

At the time of the study termination, 527 patients had been screened for eligibility at six sites. Despite constant attempts to increase recruitment rates at the participating sites, randomization was stopped after 130 patients were enrolled, 109 (84%) of them at the sponsor-delegated person’s site in Munich. The most common reasons for screening failure were failure to meet criteria for enrolment such as low baseline severity level with respect to VM-related attack incidence prior to enrolment, refusal to provide informed consent, excluded comorbidities, and ongoing beta-blocker therapy due to indications not necessarily associated with VM.

Figure 1 illustrates the flow of patients through the trial together with patient-reported outcome specific information [20]. In total, 130 patients were allocated to either metoprolol or placebo and were included in the intention-to-treat population. The FAS population consisted of 127 patients with three patients being excluded after randomization: two patients allocated to placebo (concomitant diagnosis of BPPV causing inability to discriminate episodes caused by VM from the ones caused by BPPV) did not fulfil the major entry criterion with respect to baseline attack severity; and one patient allocated to metoprolol fulfilled a major exclusion criterion (concomitant diagnosis vestibular paroxysmia with no diary information provided). In the placebo group, nine patients did not provide any diary information compared to four patients in the metoprolol group. Within the 3-month assessment period, diary data were available for 91 out of 127 patients of the FAS sample (42 patients in the placebo group versus 49 patients in the metoprolol group). The proportion of intermittent missing information was rather low for both treatment groups whereas the proportion of monotone missing diary information, e.g. due to treatment adherence or noncompliance, was rather high. Besides, the proportion of missing diary data was higher in the placebo than in the metoprolol group (Additional file 4: Figure S2).

Table 1 gives the demographic and important clinical characteristics including the vertigo-specific quality-of-life DHI score of all randomized patients assessed at the baseline visit. Overall, 60.8% of the randomized patients were female and the median age was 44 years. The proportion of patients diagnosed with definite (as opposed to probable) VM was 61.5%, with 64.6% in the metoprolol group being slightly higher than 58.5% in the placebo group. Overall, both groups were similar to each other in respect of demographics and baseline clinical patient characteristics. Pre-randomization attack frequency with respect to the domains of vertigo and migraine was not documented although considered as a key inclusion criterion. No information with regard to disease duration or age at onset was available.

**Table 1:** Baseline and disease characteristics of the intention-to-treat sample

Characteristics	Placebo (*n* = 65)	Metoprolol (*n* = 65)
Demographics		
Age (years)		
Mean (SD)	42.8 (14.3)	44.4 (14.2)
Median (range)	44.0 (19–70)	45.0 (19–75)
Male sex		
*n* (%)	29 (44.6)	22 (33.8)
VM diagnostic criteria		
Probable VM, *n* (%)	27 (41.5)	23 (35.4)
Definite VM, *n* (%)	38 (58.5)	42 (64.6)
General physical examination		
Body mass index (kg/m^2^)		
Mean (SD)	26.4 (5.5)	25.9 (3.9)
Median (range)	25.3 (17.6–46.6)	26.0 (17.5–38.1)
Missing, *n* (%)	5 (7.7)	6 (9.2)
Systolic blood pressure (mmHg)		
Mean (SD)	134.1 (18.6)	136.8 (16.3)
Median (range)	130.0 (100.0–188.0)	136.0 (109.0–180.0)
Missing, *n* (%)	9 (13.8)	6 (9.2)
Diastolic blood pressure (mmHg)		
Mean (SD)	85.8 (10.1)	85.5 (9.7)
Median (range)	86.0 (66.0–108.0)	84.0 (68.0–107.0)
Missing, *n* (%)	9 (13.8)	6 (9.2)
Heart rate (bpm)		
Mean (SD)	74.8 (10.0)	72.4 (10.3)
Median (range)	73.0 (60.0–100.0)	73.0 (54.0–100.0)
Missing, *n* (%)	8 (12.3)	6 (9.2)
DHI total score^a^		
Mean (SD)	1.7 (0.8)	1.6 (0.7)
Median (range)	1.7 (0.4–3.5)	1.5 (0.4–3.1)
Missing, *n* (%)	1 (1.5)	2 (3.1)
Physical examination		
Cranial nerves: head-shaking nystagmus		
Patients with nystagmus, *n* (%)	4 (6.2)	4 (6.2)
Missing, *n* (%)	5 (7.7)	6 (9.2)
Coordination: Romberg’s test		
Patients with instability, *n* (%)	3 (4.6)	4 (6.2)
Missing, *n* (%)	2 (3.1)	0 (0.0)
Neuro-orthoptic examinations		
Smooth pursuit eye movement		
Saccaded, *n* (%)	25 (41.5)	29 (44.6)
Missing, *n* (%)	2 (1.5)	0 (0.0)
Absolute SVV deviation (°)		
Mean (SD)	0.5 (1.2)	0.4 (1.2)
Median (range)	0.0 (0.0–5.0)	0.0 (0.0–6.0)
Missing, *n* (%)	3 (4.6)	0 (0.0)
Gaze-evoked nystagmus		
*n* (%)	7 (10.8)	14 (21.5)
Missing, *n* (%)	2 (3.1)	0 (0.0)
Nystagmus in the scanning laser ophthalmoscope		
*n* (%)	8 (12.3)	3 (4.6)
Missing, *n* (%)	12 (18.5)	9 (13.8)
Disturbed fixation suppression		
*n* (%)	6 (9.2)	3 (4.6)
Missing, *n* (%)	4 (6.2)	1 (1.5)
Oculography		
Spontaneous nystagmus (°/s)		
Velocity = 0, *n* (%)	47 (72.3)	48 (73.8)
Velocity ≥1, *n* (%)	16 (24.5)	16 (24.5)
Velocity ≥3, *n* (%)	1 (1.5)	1 (1.5)
Missing, *n* (%)	2 (3.1)	1 (1.5)
Gaze-evoked nystagmus (°/s)		
Velocity = 0, *n* (%)	14 (21.5)	7 (10.8)
Velocity ≥1, *n* (%)	43 (66.1)	49 (75.3)
Velocity ≥3, *n* (%)	6 (9.2)	10 (15.3)
Missing, *n* (%)	8 (12.3)	9 (13.8)
Bithermal caloric testing (normalized right–left difference (%) according to Jongkees’ formula^b^)		
Mean (SD)	−8.5 (20.5)	−6.9 (21.9)
Median (range)	−10.8 (−58.3 to 73.0)	−5.9 (−82.9 to 71.4)
Missing, *n* (%)	6 (9.2)	5 (7.7)

*SD* standard deviation, *SVV* subjective visual vertical, *VM* vestibular migraine

^a^ Dizziness handicap inventory (DHI); high score indicates high impairment; range of possible total scores, 0–100; mean total score (range 0–4) indicates averaging for the number of answered questions (see Additional file 4)

^b^ Jongkees’ formula = 100·(|RC| + |RW| – (|LC| + |LW|)) / (|RC| + |LC| + |RW| + |LW|) $\frac{\left(\left|\mathrm{RC}\right|+\left|\mathrm{RW}\right|-\left(\left|\mathrm{LC}\right|+\left|\mathrm{LW}\right|\right)\right)}{\left(\left|\mathrm{RC}\right|+\left|\mathrm{LC}\right|+\left|\mathrm{RW}\right|+\left|\mathrm{LW}\right|\right)}$; where RW = right warm rinse (44 °C water), LW = left warm rinse, RC = right cold rinse, LC = left cold rinse; the condition “warm” means irrigation with 44 °C water; “cold” means irrigation with 30 °C water

#### Dosing and adherence to initial treatments

In total, 121 patients received at least one dose of the study medication. In the FAS, the median treatment duration (range) was 177 (0–203) days in the placebo group, and 178 (0–236) days in the metoprolol group (the difference in location of median duration (95% confidence interval (CI)) was −1.999 (−6.000 to 2.999; *P* = 0.451). A considerable number of patients took rescue medication on an as-needed basis. However, the proportion was comparable in both randomized groups (data not shown).

### Primary and key secondary efficacy analyses

For the FAS sample, 114 patients (54 on placebo, 60 on metoprolol) contributed data to the Poisson mixed-effects model which revealed an overall relief in vertigo-related symptoms over time in both treatment groups. The mean incidence rate of vertigo attacks in those receiving placebo was significantly reduced by a factor of 0.830 per additional month while on treatment (95% CI 0.776–0.887; *P* < 0.001). It was hypothesized that the assigned active treatment would make this overall decay rate even smaller. The corresponding estimated factor, representing the IRR compared to placebo, was 0.983 (95% CI 0.902–1.071) in those receiving metoprolol; no evidence for a treatment-by-time interaction was found (global testing, likelihood-ratio test, *P* = 0.696) indicating no statistically significant difference in the monthly incidence rates for vertigo attacks between both groups. Table 2 displays the results for months 4, 5 and 6, representing the pre-specified 3-month time period of primary interest to assess treatment effectiveness. Within month 6, the marginal mean incidence rate per 30 days for vertigo attacks was 3.097 (95% CI 1.914–4.281) for patients receiving placebo versus 2.796 (95% CI 1.792–3.800) for those receiving metoprolol. Similarly, for the per-protocol sample (comprising of 89 patients; 41 receiving placebo), the overall decay rate was 0.848 (95% CI 0.795–0.904; *P* < 0.001) and the IRR was estimated to be 0.978 (95% CI 0.901–1.061; *P* = 0.593) indicating a lack of a beneficial treatment effect.

**Table 2:** Summary of diary-based primary and secondary end points for the full analysis set population

	*N*^a^	Placebo	Metoprolol
Primary end point			
Vertigo attacks, monthly^b^ incidence rates (95% CI)^a^	114		
Month 4		4.499 (3.295–5.704)	4.202 (3.138–5.267)
Month 5		3.733 (2.527–4.939)	3.428 (2.384–4.471)
Month 6		3.097 (1.914–4.281)	2.796 (1.792–3.800)
Decay rate (95% CI), *P* value		0.830 (0.776–0.887), <0.001	
IRR (95% CI), *P* value		0.983 (0.902–1.071), 0.696	
Secondary end points			
Vertigo days, monthly^b^ incidence rates (95% CI)^a^	114		
Month 4		6.757 (5.067–8.447)	5.278 (3.999–6.557)
Month 5		5.881 (4.126–7.637)	4.319 (3.070–5.569)
Month 6		5.119 (3.326–6.912)	3.534 (2.334–4.735)
Decay rate (95% CI), *P* value		0.870 (0.821–0.923), <0.001	
IRR (95% CI), *P* value		0.940 (0.869–1.017), 0.125	
Mean monthly^b^ MHDs^c^	91		
Months 4–6		2.400 (1.410–4.410)	2.505 (1.488–4.215)
IRR (95% CI), *P* value		1.048 (0.482–2.250), 0.904	

*CI* confidence interval

^a^ Primary efficacy analysis by a Poisson generalized linear mixed model (with random intercept and slope) based on the whole 6-month treatment period; assumption is maximal effect of intervention during the pre-specified 90-day assessment period in months 4–6; analysis of vertigo day rates performed as a supplementary analysis

^b^ Mean incidence rates per 30 days derived by a model-based approach assuming missingness at random; pre-specified time period of primary interest months 4–6; reference group = placebo

^c^ Migraine headache days (MHDs): rates and incidence rate ratios (IRRs) are estimated from a negative binomial generalized linear model based on the aggregated MHD data reported within months 4–6 only (91 patients contributing MHD-related diary documentation)

**Fig. 2:**
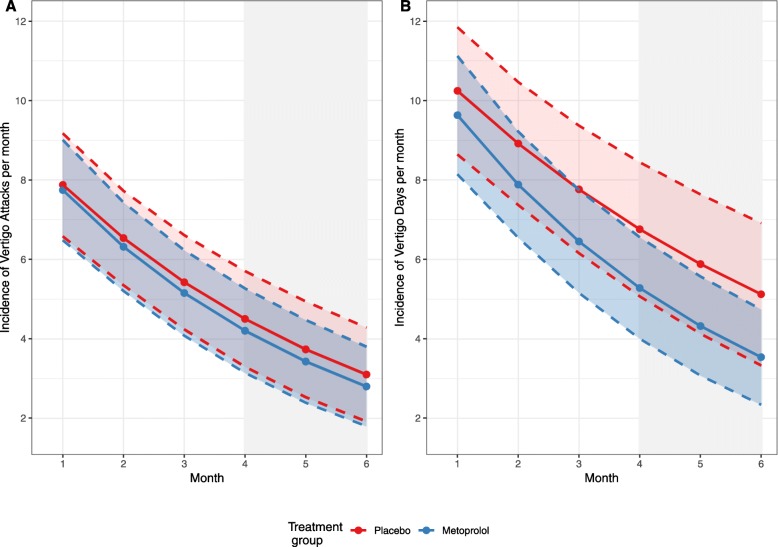
Predicted marginal means (with pointwise 95% confidence intervals) for (**a**) the incidence of vertigo attacks (primary efficacy outcome) and (**b**) vertigo days per 30 days during the 6-month treatment period (full analysis set population). A Poisson random intercept and slope model was applied for the principal analysis. The grey shaded area represents the 90-day time period of primary interest comprising of estimated monthly incidences rates for months 4 to 6

Considering the robustness of the primary result, a supplementary analysis for vertigo days was conducted in line with the primary efficacy analysis. For the FAS sample, the mean monthly incidence of vertigo days was reduced by 13% per additional month on placebo treatment, i.e. by a factor of 0.870 (95% CI 0.821–0.923; *P* < 0.001). However, no superiority of metoprolol compared to placebo was found, with an estimated IRR of 0.940 (95% CI 0.869 to 1.017; *P =* 0.125). Figure 2a depicts the estimated monthly incidence rates for vertigo attacks during the whole 6-month treatment period for the placebo and metoprolol group. Figure 2b shows the estimated monthly incidence rates over time for the key secondary outcome of vertigo days. During the 3-month assessment period (months 4 to 6) the mean monthly incidence for MHD was similar in the placebo and metoprolol group. The corresponding IRR defining the treatment effect estimate was 1.048 (95% CI 0.482–2.250; *P* = 0.904).

Table 3 summarizes the results for planned key secondary efficacy outcome analyses at the pre-specified time point of month 6. The DHI mean total score evaluating the self-perceived handicapping effects imposed by VM remained fairly stable at the end of the treatment period compared to the score measured at the baseline visit. The complete case analysis of covariance revealed no evidence for a between-treatment difference in mean change scores (Δ = −0.079 (95% CI −0.360 to 0.201; *P* = 0.577)). No statistically significant and clinically meaningful difference between placebo and metoprolol could be detected for smooth pursuit eye movement and SVV assessments. For both clinician-reported end points, the chance of achieving treatment response, i.e. a change from ‘abnormal’ at baseline to ‘normal’ at month 6, did not differ between both groups. For smooth pursuit eye movement, the odds ratio (OR) was estimated to be 1.483 (95% CI 0.454–5.277; *P* = 0.520), and, for SVV, the OR was 0.413 (95% CI 0.055–2.235; *P* = 0.322) for metoprolol compared with placebo. Hence, patients assigned to metoprolol did not achieve superior patient-reported and clinician-reported outcomes compared to those assigned to placebo treatment, suggesting robustness of the principal result.

**Table 3:** Key secondary outcome results (least square mean change difference or odds ratio)^a^

Secondary End points	*N*^b^	Placebo	Metoprolol
DHI mean total score	91		
LS mean change^c^ (95% CI)		0.159 (−0.252 to 0.570)	0.080 (−0.310 to 0.470)
Difference vs. placebo (95% CI)		−0.079 (−0.360 to 0.201)	
*P* value		0.577	
Pursuit eye movement	92		
Patients achieving response^d^, *n* (%)		5 (11.6)	8 (16.3)
OR (95% CI)		0.132 (0.045–0.305)	0.195 (0.092–0.416)
Difference vs. placebo, OR (95% CI)		1.483 (0.454–5.277)	
*P* value		0.520	
SVV	90		
Patients achieving response^d^, *n* (%)		4 (9.5)	2 (4.2)
OR (95% CI)		0.105 (0.032–0.262)	0.043 (0.012–0.179)
Difference vs. placebo, OR (95% CI)		0.413 (0.055–2.235)	
*P* value		0.322	

*CI* confidence interval

^a^ Least square (LS) mean change difference (estimates derived by complete case analysis of covariance for absolute change scores) or odds ratio (OR; estimates derived by logistic regression (unadjusted)); analysis of absolute change from baseline at month 6 for Dizziness Handicap Inventory (DHI) by analysis of covariance; for change state from baseline at month 6 in eye movement and subjective visual vertical (SVV) by logistic regression for the full analysis set (FAS) sample

^b^ Numbers of patients with non-missing observations for both baseline and 6-month visit (FAS population: *n* = 127; placebo *n* = 63; metoprolol *n* = 64)

^c^ Change score means difference between post-intervention score at month 6 versus baseline score; see Table 1 for description of DHI score ranges

^d^ Logistic regression for the change state in smooth pursuit eye movement or SVV between baseline and month 6 (1, change from abnormal to normal; 0, otherwise); pursuit eye movement — treatment response means change from ‘saccadic’ to ‘smooth’

### Safety and tolerability

Since metoprolol succinate is a well-established drug that has been used for many years in common diagnoses such as hypertension and episodic or chronic migraine, it was expected to be generally well tolerated here. Hence, there were no protocol-defined adverse events of special interest. No deaths or suspected unexpected serious adverse reactions occurred during the trial. Eighteen patients (nine in the placebo group and nine in the metoprolol group) reported a total of 21 SAEs over the whole 9-month study period.

Within the maximum treatment duration of 6 months, a total of 348 AEs occurred (174 in each group) for the safety population; 18.6% (11/59) of patients receiving placebo were not affected by AEs compared to 16.1% (10/62) receiving metoprolol. With respect to AE severity, the incidence was similar for both groups (AEs in the placebo group: 45.1% mild, 16.2% severe; in the metoprolol group: 42.2% mild, 14.5% severe). In both groups at least three AEs occurred for 50% of patients (placebo: 49.2% (29/59); metoprolol: 50.0% (31/62)). Fifteen patients (nine in the placebo group and six in the metoprolol group) were affected by a total of 17 SAEs while on study treatment. Two severe, treatment-emergent SAEs (one in each group: hospitalization due to diverticulitis in the placebo group and hospitalization due to migraine in the metoprolol group; both recovered) were suspected by the investigator to be causally related to treatment. One patient receiving metoprolol discontinued the treatment owing to an SAE, and seven discontinued because of non-serious AEs, as compared to two patients on placebo because of SAEs and two because of non-serious AEs. Detailed information of the frequency of AEs which occurred within the 6 months of intervention is displayed in Table 4.

**Table 4:** Safety assessment by study treatment group (safety sample) during the 6-month treatment period

Safety assessment	Placebo (*n* = 59)	Metoprolol (*n* = 62)
Deaths, *n*	0	0
Patients with SUSARs, *n*	0	0
Patients with early termination from the study due to SAEs^a^, *n* (%)	2 (3.4)	1 (1.6)
Treatment-related SAEs, *n* (%)	1 (1.7)	1 (1.6)
Patients with at least one SAE, *n* (%); total number of SAEs	8 (13.5); 10	6 (9.7); 7
Patients with early termination due to adverse events^a^, *n* (%)	4 (6.7)	8 (12.9)

Percentages (%) are based on the number of patients in the safety sample

Reasonable possibility for a causal relationship = drug-event relationship reported as “possible”, “probable”, or missing according to the adverse event case report form

^a^ Adverse event or serious adverse event (SAE) leading to treatment discontinuation according to the adverse event case report form

*SUSAR* suspected unexpected serious adverse reactions

## Discussion

VM is considered the most common neurologic cause of recurrent spontaneous vertigo episodes [32]. The main reason for the diagnostic challenge is the broad spectrum of its manifestations, e.g. episodic vestibular symptoms without typical migraine headaches, and the wide variety of ictal and interictal symptoms [33].

### Principal findings

The PROVEMIG trial proved not to be feasible regarding patient recruitment and was terminated early after randomization of 130 patients, i.e. after achieving about 50% of the target enrolment. Nevertheless, there are several important findings and lessons that can be learnt from our study to inform future interventional drug trials in this patient population and to apply methods of quantitative evidence synthesis in terms of meta-analyses.

The key finding of the trial are as follows. First, in both randomized groups, patients experienced a significant reduction in the monthly incidence of vertigo attacks (according to the definition used in this study, i.e. lasting between 5 min and no longer than 72 h) of 17.0% (95% CI 11.3–22.4%) over the whole double-blind 6-month treatment period. However, prophylactic treatment with the beta blocker metoprolol was not superior to placebo in diminishing the monthly incidence of vertigo attacks over time (IRR 0.983 (95% CI 0.902–1.071)). Although the trial was prematurely ended, the 95% CIs for the estimated decay rate and IRR are rather narrow; apparently, large treatment effects are not very likely given the present results. Second, no beneficial therapeutic effect of metoprolol could be established either in the patient-reported efficacy outcomes (including the DHI total score measured by a psychometrically validated questionnaire) or in the clinical assessments. Third, the investigational drug and placebo regimens were approximately equally safe and well tolerated in the participating patients, with no unexpected safety findings.

In summary, there is no evidence from randomized controlled trials to support or refute treatment with metoprolol in patients diagnosed with VM.

### Comparison with previous literature

To our knowledge, this is the first report of a pragmatic, double-blind, randomized, placebo-controlled trial investigating the effectiveness, tolerability and safety of a prophylactic symptomatic treatment with metoprolol compared to placebo in patients with VM. The first Cochrane systematic review on this topic published in 2015 aimed to assess the effects of pharmacological agents (including beta blockers) used in the prophylactic treatment of VM-associated symptoms against placebo or no treatment (“wait-and-see”). However, the authors did not find any evidence from completed randomized controlled trials using the Bárány Society/International Headache Society diagnostic criteria, while identifying PROVEMIG as the only ongoing trial fulfilling the inclusion criteria with respect to trial design [9].

### Strengths and weaknesses

The trial population consisted of 130 patients (with 121 patients commencing the allocated intervention) selected from 527 patients screened for eligibility. Patients were diagnosed according to the established Neuhauser criteria 2001 [2]. The current International Classification of Vestibular Disorders criteria for VM were not available at that time, which are mostly similar to “definite VM” according to Neuhauser. Investigators at the participating trial sites were clinical experts in the diagnosis and treatment of vestibular disorders. Due to the complexity of the disease entity, expert knowledge with respect to diagnosis is essential in order to differentiate between VM and other diseases with spontaneous recurrent vertigo, most notably Menière’s disease [34–36]. Baseline assessments (Table 1) provide neuro-otological and ophthalmological data systematically collected from a well-defined sample of VM patients. The female preponderance in the study population was found to be 1.5:1 which is consistent with the 1.5–5:1 female-to-male ratio reported in other studies [2, 37].

The 6-month duration of treatment allowed us to ascertain whether the active agent may be distinguished from placebo by how quickly patients achieve a reduction in monthly vertigo attacks (‘speed of effect’). Since the target was comparing the benefit of two treatment policies (i.e. the drug as actually taken), all patients were allowed to take rescue medication if necessary. About 68% of patients comprising the per-protocol population were at least 3 months on treatment. Altogether, the proportion of missing data with respect to the diary-based efficacy outcomes was not higher than expected for symptomatic trials assessing the ability of an intervention to provide symptom relief from the condition.

In this trial, the experimental treatment with the beta blocker metoprolol was compared in a blinded manner with an unspecific treatment, i.e. placebo, which is assumed to cover all the unspecific effects of an intervention (e.g. patient expectations, natural course or regression-to-the-mean) [38]. The findings for patients receiving placebo may not fully reflect the natural course of VM which has been reported to vary over time.

Our study has certain limitations. Faced with persisting recruitment difficulties, the trial was not successful in reaching the target sample size. Therefore, we cannot perform confirmatory analyses, but provide 95% CIs for estimators of treatment effectiveness. One factor that led to delays and early trial closure was the number of screening failures being higher than expected. Reasons for the poor participant accrual were multifactorial and included unwillingness to accept the underlying intervention and failure to meet eligibility criteria (in particular, a low baseline frequency with respect to attacks of VM, or comorbidities being contraindications for metoprolol). Furthermore, the number of participating trial sites was lower than anticipated. With 84% of 130 randomized patients recruited at a single site, the highly specialized dizziness unit at the Ludwig Maximilians University, Munich, which attracts patients from all over Germany, our findings may not be applicable to all German patients with VM. Due to the considerable overlap of the two disorders of VM and Menière’s disease, the study population might be contaminated.

Since VM is a complex and relatively new single disease entity, clinically meaningful patient-centred primary efficacy outcomes are still being debated. In this trial, a patient dizziness diary routinely used in clinical practice for diagnosis at the site of the principal investigator was adapted for the clinical trial setting. Paper-based daily diaries are prone to errors or being lost and have no simple methods for backup and reconstruction of information. If paper-based symptom diaries are applied for tracking recurrent events and to understand longitudinal relationships over time in confirmatory clinical trials, patient recordings have to be reviewed by the site personnel for accuracy and interpretability of reported symptoms in order to derive rigidly defined primary efficacy end points. To face this dilemma, further research is warranted to define responsive patient-centred outcomes for both outcome domains (vertigo and migraine headache) that have been shown to be important to patients and clinicians to allow informed decision making. Since patients with VM experience a wide variety of ictal and interictal symptoms, more effort should be made in investigating whether established outcome measurement instruments for both domains are appropriate for this target population, and in evaluating the quality of these instruments (primarily with respect to responsiveness to change) [39]. As such, the validated scores derived from the self-reported DHI questionnaire together with self-administered migraine-specific quality-of-life questionnaires such as the Migraine Disability Assessment or the Headache Impact Test might be used to evaluate the treatment benefit while reducing the documentation burden for the patient compared to a diary-based measurement of disability which could be essential for long-term comparative effectiveness trials [40–44].

The following points should be mentioned with respect to the PROVEMIG diary (Additional file 3):

1. With headache as a co-primary end point, migraine headache symptoms should have been assessed in a dedicated column on the diary form instead of one of the potential accompanying symptoms for vertigo episodes.
2. A simple visual analogue scale integrated in the diary might have helped patients, leading to a better rating with respect to the severity of symptoms (primarily with a lower rate of missing data).
3. Daily diary entries have to be regularly monitored by the site personnel during study visits (in order to distinguish the absence of episodes from non-compliance with diary maintenance). In the case of electronic diaries which could be used in future trials (ideally based on an app which can remind the patient on a daily basis to update) this can be continuously done online and assisted by algorithms, which potentially would improve data quality provided that they give useful guidance rather than bossing around the patient leaving them frustrated.
4. Regardless if a paper-based or e-diary is applied, scoring algorithms to retrieve the pre-specified efficacy end points have to be established and validated. Without doing so, we would not have been able to carry out meaningful longitudinal analyses of the PROVEMIG diary data.

## Conclusions

It is of the utmost importance to develop a core outcome set for this complex vestibular disease comprising of symptoms attributable to both vertigo and migraine headache, with the aim of reducing the documentation burden for the trial participants in long-term trials and defining clinically meaningful, patient-centred efficacy end points that are sensitive to change and that are reproducible [45–47]. A strong placebo response was observed in the PROVEMIG trial which is well known from other trials of migraine treatment [48].

For future phase III trials, a more efficient site set-up and improved recruitment methods would seem to be appropriate. Furthermore, study site personnel should aim to follow patient retention strategies to ensure that participants, once recruited, are engaged clinically and followed up as completely as possible to avoid non-compliance with diary maintenance in particular. Additional multicentre, randomized, placebo-controlled trials are needed to replicate these findings and to explore subgroups of patients reporting response to antihypertensive drugs.

## Supplementary information

**Additional file 1.** Consolidated Standards of Reporting Trials (CONSORT) 2010 checklist of information to include when reporting a randomized trial.

**Additional file 2.** Original study protocol.

**Additional file 3.** Patient symptom diary (original German version; word-for-word English translation).

**Additional file 4.** Procedural and statistical methods.

## Data Availability

Data cannot be shared publicly because participants did not explicitly consent to the sharing of their data as per the European Union’s General Data Protection Regulation and the corresponding German privacy laws. Data are available through the Research Ethics Board of the Ludwig-Maximilians-Universität, Munich, Germany, for researchers who meet the criteria for access to confidential data. Please address requests to ethikkommission@med.uni-muenchen.de (ethics committee of the LMU Munich) and to the biostatistician at mansmann@ibe.med.uni-muenchen.de.

## Abbreviations

AE: Adverse event
BPPV: Benign paroxysmal positional vertigo
CI: Confidence interval
DHI: Dizziness Handicap Inventory
FAS: Full analysis set
IRR: Incidence rate ratio
MAR: Missing at random
MHD: Migraine headache day
OR: Odds ratio
SAE: Serious adverse event
SVV: Subjective visual vertical
VM: Vestibular migraine

## References

[1] Headache Classification Committee of the International Headache Society (IHS) (2018). The international classification of headache disorders, 3rd edition. Cephalalgia.

[2] Neuhauser H, Leopold M, von Brevern M, Arnold G, Lempert T (2001). The interrelations of migraine, vertigo, and migrainous vertigo. Neurology.

[3] Lempert T, Olesen J, Furman J, Waterston J, Seemungal B, Carey J, Bisdorff A, Versino M, Evers S, Newman-Toker D (2012). Vestibular migraine: diagnostic criteria. J Vestib Res.

[4] Furman JM, Marcus DA, Balaban CD (2003). Migrainous vertigo: development of a pathogenetic model and structured diagnostic interview. Curr Opin Neurol.

[5] Marcus DA, Kapelewski C, Rudy TE, Jacob RG, Furman JM (2004). Diagnosis of migrainous vertigo: validity of a structured interview. Med Sci Monit.

[6] Neuhauser HK (2007). Epidemiology of vertigo. Curr Opin Neurol.

[7] Formeister EJ, Rizk HG, Kohn MA, Sharon JD (2018). The epidemiology of vestibular migraine: a population-based survey study. Otol Neurotol.

[8] Strupp Michael, Versino Maurizio, Brandt Thomas (2010). Vestibular migraine. Handbook of Clinical Neurology.

[9] Maldonado Fernandez M, Birdi JS, Irving GJ, Murdin L, Kivekas I, Strupp M. Pharmacological agents for the prevention of vestibular migraine. Cochrane Database Syst Rev. 2015;(6):Cd010600. 10.1002/14651858.CD010600.pub2 PMC6494480 26093662

[10] Lauritsen CG, Marmura MJ (2017). Current treatment options: vestibular migraine. Curr Treat Options Neurol.

[11] Stolte B, Holle D, Naegel S, Diener HC, Obermann M (2015). Vestibular migraine. Cephalalgia.

[12] Bisdorff AR. Treatment of migraine related vertigo with lamotrigine an observational study. Bull Soc Sci Med Grand Duche Luxemb. 2004;2:103–8. 15641316

[13] Carmona S, Settecase N (2005). Use of topiramate (topamax) in a subgroup of migraine-vertigo patients with auditory symptoms. Ann N Y Acad Sci.

[14] Reploeg MD, Goebel JA (2002). Migraine-associated dizziness: patient characteristics and management options. Otol Neurotol.

[15] Lepcha A, Amalanathan S, Augustine AM, Tyagi AK, Balraj A (2014). Flunarizine in the prophylaxis of migrainous vertigo: a randomized controlled trial. Eur Arch Otorhinolaryngol.

[16] Salviz M, Yuce T, Acar H, Karatas A, Acikalin RM (2016). Propranolol and venlafaxine for vestibular migraine prophylaxis: a randomized controlled trial. Laryngoscope.

[17] Evers S, Afra J, Frese A, Goadsby PJ, Linde M, May A, Sandor PS (2009). European Federation of Neurological S: EFNS guideline on the drug treatment of migraine—revised report of an EFNS task force. Eur J Neurol.

[18] Diener HC, Katsarava Z, Limmroth V (2008). Current diagnosis and treatment of migraine. Ophthalmologe.

[19] Moher D, Hopewell S, Schulz KF, Montori V, Gøtzsche PC, Devereaux PJ, Elbourne D, Egger M, Altman DG (2010). CONSORT 2010 explanation and elaboration: updated guidelines for reporting parallel group randomised trials. BMJ.

[20] Calvert M, Blazeby J, Altman DG, Revicki DA, Moher D, Brundage MD, Group CP (2013). Reporting of patient-reported outcomes in randomized trials: the CONSORT PRO extension. JAMA.

[21] Butcher NJ, Monsour A, Mew EJ, Szatmari P, Pierro A, Kelly LE, Farid-Kapadia M, Chee-a-tow A, Saeed L, Monga S (2019). Improving outcome reporting in clinical trial reports and protocols: study protocol for the Instrument for reporting Planned Endpoints in Clinical Trials (InsPECT). Trials.

[22] Jacobson GP, Newman CW (1990). The development of the Dizziness Handicap Inventory. Arch Otolaryngol Head Neck Surg.

[23] Kurre A, van Gool CJ, Bastiaenen CH, Gloor-Juzi T, Straumann D, de Bruin ED (2009). Translation, cross-cultural adaptation and reliability of the German version of the dizziness handicap inventory. Otol Neurotol.

[24] CHMP. ICH E9 (R1) Addendum on estimands and sensitivity analysis in clinical trials to the guideline on statistical principles for clinical trials. EMA/CHMP/ICH/436221/201, Step 4 (Final Version adopted on 20 Nov 2019). European Medicines Agency, Committee for Medicinal Products for Human Use (CHMP); 2019. https://database.ich.org/sites/default/files/E9-R1_Step4_Guideline_2019_1203.pdf. Accessed 17 Dec 2019.

[25] Molenberghs G, Thijs H, Jansen I, Beunckens C, Kenward MG, Mallinckrodt C, Carroll RJ (2004). Analyzing incomplete longitudinal clinical trial data. Biostatistics.

[26] O'Kelly M, Ratitch B. Clinical trials with missing data: a guide for practitioners. Chichester: Wiley; 2014.

[27] White IR, Carpenter J, Horton NJ (2012). Including all individuals is not enough: lessons for intention-to-treat analysis. Clin Trials.

[28] CHMP. Guideline on clinical investigation of medicinal products for the treatment of migraine. In: CPMP/EWP/788/2001 Rev 1. European Medicines Agency, Committee for Medicinal Products for Human Use (CHMP); 2007. https://www.ema.europa.eu/en/documents/scientific-guideline/guideline-clinical-investigation-medicinal-products-treatment-migraine_en.pdf. Accessed 17 Dec 2019.

[29] R Development Core Team (2019). R: a language and environment for statistical computing.

[30] Bates D, Maechler M, Bolker B, Walker S (2014). lme4: Linear mixed-effects models using Eigen and S4, R package version 1.1–7.

[31] Bates D, Maechler M, Bolker B, Walker S (2014). Fitting linear mixed-effects models using lme4.

[32] Obermann M (2017). Editorial: vestibular migraine. Front Neurol.

[33] Beh Shin C., Masrour Shamin, Smith Stacy V., Friedman Deborah I. (2019). The Spectrum of Vestibular Migraine: Clinical Features, Triggers, and Examination Findings. Headache: The Journal of Head and Face Pain.

[34] Radtke Andrea, Neuhauser Hannelore, von Brevern Michael, Hottenrott Tilman, Lempert Thomas (2011). Vestibular migraine – validity of clinical diagnostic criteria. Cephalalgia.

[35] Strupp M, Lopez-Escamez JA, Kim JS, Straumann D, Jen JC, Carey J, Bisdorff A, Brandt T (2016). Vestibular paroxysmia: diagnostic criteria. J Vestib Res.

[36] Lopez-Escamez JA, Dlugaiczyk J, Jacobs J, Lempert T, Teggi R, von Brevern M, Bisdorff A (2014). Accompanying symptoms overlap during attacks in Meniere's disease and vestibular migraine. Front Neurol.

[37] Lempert T (2013). Vestibular migraine. Semin Neurol.

[38] Wood L, Egger M, Gluud LL, Schulz KF, Jüni P, Altman DG, Gluud C, Martin RM, Wood AJG, Sterne JAC (2008). Empirical evidence of bias in treatment effect estimates in controlled trials with different interventions and outcomes: meta-epidemiological study. BMJ.

[39] Prinsen CA, Vohra S, Rose MR, Boers M, Tugwell P, Clarke M, Williamson PR, Terwee CB (2016). How to select outcome measurement instruments for outcomes included in a “Core Outcome Set” — a practical guideline. Trials.

[40] Benz T, Lehmann S, Gantenbein AR, Sandor PS, Stewart WF, Elfering A, Aeschlimann AG, Angst FJH. Translation, cross-cultural adaptation and reliability of the German version of the migraine disability assessment (MIDAS) questionnaire. 2018;16(1):42. 10.1186/s12955-018-0871-5 PMC5845367 29523138

[41] Yang M, Rendas-Baum R, Varon SF, Kosinski M (2011). Validation of the Headache Impact Test (HIT-6) across episodic and chronic migraine. Cephalalgia.

[42] Martin M, Blaisdell B, Kwong JW, Bjorner JB (2004). The short-form Headache Impact Test (HIT-6) was psychometrically equivalent in nine languages. J Clin Epidemiol.

[43] Park JW, Shin HE, Kim JS, Lee KS (2008). Assessing migraine disability by diary-based measurement: relationship to the characteristics of individual headache attacks. Eur J Neurol.

[44] Stewart WF, Lipton RB, Kolodner KB, Sawyer J, Lee C, Liberman JN (2000). Validity of the Migraine Disability Assessment (MIDAS) score in comparison to a diary-based measure in a population sample of migraine sufferers. Pain.

[45] Kirkham JJ, Davis K, Altman DG, Blazeby JM, Clarke M, Tunis S, Williamson PR (2017). Core Outcome Set-STAndards for Development: the COS-STAD recommendations. PLoS Med.

[46] Smith H, Horobin A, Fackrell K, Colley V, Thacker B, Hall DA (2018). Defining and evaluating novel procedures for involving patients in Core Outcome Set research: creating a meaningful long list of candidate outcome domains. Res Involv Engagem.

[47] Williamson PR, Altman DG, Bagley H, Barnes KL, Blazeby JM, Brookes ST, Clarke M, Gargon E, Gorst S, Harman N (2017). The COMET handbook: version 1.0. Trials.

[48] Antonaci F, Chimento P, Diener HC, Sances G, Bono G (2007). Lessons from placebo effects in migraine treatment. J Headache Pain.

