# Supplementary material for: Results and lessons learnt from a randomized controlled trial: prophylactic treatment of vestibular migraine with metoprolol (PROVEMIG)
Source: Trials. 2019 Dec 30;20:813. doi: 10.1186/s13063-019-3903-5 (PMC6937687; doi:10.1186/s13063-019-3903-5)
Supplement: Supplementary file 2 — Additional file 2. Original study protocol. [file 13063_2019_3903_MOESM2_ESM.pdf]

Clinical Trial Protocol

Prophylactic treatment of vestibular migraine with metoprolol: a double-blind, placebo-controlled trial

Version V 2.1, 19 December 2011

**Sponsor / Sponsor-delegated person (SDP):**	Hospital of the University of Munich, represented by<br>Prof. Dr. med. Burkhard Göke<br>Prof. Dr. med. Michael Strupp (SDP)<br>Marchioninistr. 15<br>81377 Munich
**Coordinating Investigator** (Leiter der klinischen Prüfung according to German Drug Law § 42)**:**	Prof. Dr. med. Michael Strupp<br>Integrated Center for Research and Treatment of Vertigo, Balance and Ocular Motor Disorders (IFB), University of Munich<br>Marchioninistr. 15<br>D-81377 Munich
**Representative of Coordinating Investigator:**	PD Dr. med. Klaus Jahn<br>Department of Neurology<br>Hospital of the University of Munich<br>Marchioninistr. 15<br>D-81377 Munich
**Trial Design:**	Multicenter, national, randomized, double-blind, placebo-controlled, two-arm parallel-group, efficacy of treatment trial
**Coordinating Research Center of the Hospital:**	CSC[LMU], Clinical Study Center<br>Max-Lebsche-Platz 32<br>D-81377 Munich
**Trial Short Title**	PROVEMIG
**Trial Code**	VMMET009
**EudraCT No.:**	2009-013701-34
**Authors:**	Dipl.-Stat. Christine Adrion, MPH, Dr. med. Otmar Bayer, MPH, Prof. Dr. rer. nat. Hans-Helge Müller, Dr. med. Roman Schniepp, Prof. Dr. med. Michael Strupp, Dr. med. Anne Wiermann

Investigator Agreement Page

Please provide institution´s /
clinic´s stamp here

By my signature below, I confirm that I have read, understood and agree to adhere to all conditions, instructions and restrictions as specified in this Clinical Trial Protocol.

I will discuss the Clinical Trial Protocol in detail with my colleagues and ensure that they are comprehensively informed about the trial compound / preparation and the execution of the clinical trial.

I confirm that I and my colleagues will conduct this clinical trial in compliance with the Declaration of Helsinki, the ICH-GCP guidelines, and that I will abide by the national laws and regulations.

Furthermore I and my colleagues commit ourselves not to commence subject enrollment before the authorization of the authorities and acceptance by the relevant and responsible Ethics Committee.

I recognize that any changes in the protocol must be approved by the Sponsor / Sponsor Delegated Person (SDP), the Coordinating Investigator ("Leiter der klinischen Prüfung / LKP" in accordance with the German Drug Law), the Ethics Committee and, if applicable, the respective authority before implementation except when necessary to eliminate hazards to the subjects or when changes involve only logistical or administrative aspects of the clinical trial.

Under my supervision I will have copies of this Clinical Trial Protocol and possible updates as well as access to all information regarding the carrying out of this clinical trial put at the disposal of my colleagues; in particular I will promptly forward all information from the Sponsor / Sponsor Delegated Person (SDP) on pharmaceutical safety (SUSAR) to my colleagues.

I agree to ensure that the confidential information contained in this document will not be used for any purpose other than the evaluation or conduct of the clinical investigation without prior written consent of the Sponsor / Sponsor Delegated Person (SDP).

The investigational medicinal products will be used only for the purpose of the clinical trial.

Principal investigator:

Name, first name (print)	Signature	Date

Table of Content

Page

Protocol Approval Signatures……………………………………………………………………………………i

Investigator Agreement Page…………………………………………………………………………………ii

1 Clinical Trial Synopsis.. 5

2 Schedule of Activities 11

3 Abbreviations.. 12

4 Trial Administration structure.. 13

5 Introduction.. 16

5.1 Background.. 16

5.2 Trial Rationale.. 17

5.3 Side effects and Risk Benefit Assessment.. 17

5.3.1 Side effects.. 17

5.3.2 Risk Benefit Assessment.. 20

6 Trial Objectives and Endpoints.. 21

6.1 Primary Objective.. 21

6.2 Primary Endpoint.. 21

6.3 Secondary Objective.. 21

6.4 Secondary Endpoints.. 21

7 Trial Design.. 21

7.1 Trial Design.. 21

7.2 Number of subjects.. 23

7.3 Time Schedule.. 23

8 Trial Population and Selection Criteria.. 23

8.1 Gender Distribution.. 23

8.2 Inclusion Criteria.. 23

8.3 Exclusion Criteria.. 24

8.4 Subject Information and Recruitment 25

8.5 Randomization 25

9 Investigational Medicinal Product (IMP).. 25

9.1 Specification of IMP.. 25

9.2 Packaging and Labeling of IMP....26
9.3 Storage requirements....27
9.4 Handling of IMP at the Site and Drug Accountability....27
9.5 Dosage, Mode of Application and Dose Schedule....27
9.6 Subject Compliance....28
9.7 Return and Disposal of IMP....28
9.8 Blinding and Emergency Codes....28
9.9 Unblinding....28
9.10 Prior and Concomitant Therapy / Medication....29
9.10.1 Previous or concomitant therapy / medication....29
9.10.2 Prohibited therapy / concomitant medication....29
9.10.3 Rescue / Escape / Salvage Therapy....29

10 Trial Procedures....30
10.1 Methods of Assessment....30
10.1.1 Oculography (Electro- or Videooculography)....30
10.1.2 Vestibular Evoked Myogenic Potential Testing (VEMP)....30
10.1.3 Subjective visual vertical (SVV)....30
10.1.4 Dizziness handicap inventory (DHI) Questionnaire....31
10.1.5 Dizziness diary....31
10.1.6 Laboratory examinations / Biological Specimens....31
10.1.7 Electrocardiography....32
10.2 Time schedule of Measurements....32
10.2.1 Screening period....32
10.2.2 Treatment period....32
10.2.3 Follow-up period....33
10.2.4 Final Visit (Visit 8, End of study visit or premature termination)....33
10.2.5 End of the trial....33

11 Safety Data Documentation and Reporting....34
11.1 Definitions....34
11.2 Criteria to be evaluated by the investigator (1st assessment)....35
11.2.1 Assessment of Intensity....35
11.2.2 Assessment of Seriousness....35

11.2.3 Assessment of Causality 35
11.3 Criteria to be evaluated by the Sponsor-Delegated Person (2nd assessment).......................35
11.4 Documentation and Reporting of Adverse Events..35
11.5 Documentation and Reporting of Serious Adverse Events..36
11.5.1 Initial reporting of SAEs...36
11.5.2 Reporting to the authorities and ethics committees...36
11.6 Pregnancy...36
11.6.1 Actions to be taken if pregnancy occurs to female subjects or partners of male subjects....37

12 Data Safety Monitoring Board...38

13 Statistic and Analysis...38
13.1 Trial Design..38
13.2 Target Variable/Endpoints...38
13.3 Statistical Analyses..38
13.4 Interim Analysis..39
13.5 Modifications of the Statistical Design for Confirmatory Analysis..39
13.6 Sample Size Calculation...39
13.7 Populations included in the Analysis..40
13.8 Protocol Violations..40
13.8.1 Handling of Drop-outs, Withdrawal, and Missing Data..41

14 Data Collection, Handling and Record Keeping 41
14.1 Data Management...41
14.2 Data Coding...41
14.3 Documentation of Trial Data...42
14.3.1 Documentation of Trial Data in the Medical Record...42
14.3.2 Case Report Form (CRF)..42
14.4 Investigator Site File...42
14.5 Archiving..42
14.3.1 Sponsor...42
14.5.1 Investigator...43

15 Reporting...43
15.1 Statistical Report...43

16 Definition of End of Trial....43
16.1 Regular End of the Trial....43
16.2 Termination of the Trial for Individual Subjects....43
16.2.1 Termination by the Subject....44
16.2.2 Termination by the Investigator....44
16.3 Termination of One of the Treatment Arms or the Entire Trial....44
16.4 Termination of the Trial in Individual Sites....45
17 Monitoring, Audits and Inspections....45
17.1 Monitoring....45
17.2 Source Data Verification (SDV)....46
17.3 Audits and Inspections....46
18 Ethics and Good Clinical Practice....47
18.1 Responsibilities of the Sponsor....47
18.2 Responsibilities of the Investigator....47
18.3 Ethics Committee and Competent Authority(ies)....47
18.4 Compliance with the Protocol....48
18.5 Notification of General Amendments to the Protocol....48
18.6 Notification of the end of the trial....49
18.7 Annual Safety Report....49
18.8 Subject Information and Informed Consent....49
18.9 Subject Insurance....49
18.10 Data Protection and Subject Confidentality....50
18.11 Financing of the Trial....50
18.11.1 Trial Agreement / Investigator Compensation....50
18.11.2 Reimbursement of Subjects....50
19 Trial Reports....51
20 Publication....51
20.1 Publication Policy....51
21 References....52
22 Questionnaire: Dizziness Handicap Inventory (DHI)....54

1 Clinical Trial Synopsis

**Clinical trial title**	Prophylactic treatment of vestibular migraine with metoprolol: a double-blind, placebo-controlled trial
**Phase of trial**	III
**Trial short title**	PROVEMIG
**Trial Code**	VMMET009
**EudraCT No.**	2009-013701-34
**Investigational medicin-al product, Dose and Mode of Application**	Trade Name: Beloc Zok mite®<br>Substance: Metoprolol succinate<br>Manufacturer: Astra Zeneca GmbH<br>Packaging and Labeling: Pharmacy of the University Hospital Heidelberg, Im Neuenheimer Feld 670, D-69120 Heidelberg<br>Dose: 47.5 mg once daily during the first week, 95 mg after the first week, after completion of the treatment 47.5 mg per day for two weeks before stopping using<br>Mode of application: oral<br>Duration of treatment: 6 month
**Comparative Drug, Dose and Mode of Application**	Placebo<br>Manufacturer: Pharmacy of the University Hospital Heidelberg, Im Neuenheimer Feld 670, D-69120 Heidelberg<br>Dose: once daily<br>Mode of Application: oral<br>Duration of Treatment: 6 month
**Trial Population** (or indication)	Male and female subjects with vestibular migraine
**Trial Design**	Multicenter, national, randomized, double-masked, placebo-controlled, double-blind, two-arm, parallel-group efficacy of treatment study
**Trial Objectives**	Primary Objective:<br>To demonstrate the superiority of Metoprolol Succinate treatment regarding the number of vertigo attacks and the number of headache attacks per month compared to placebo.<br>Secondary Objective:<br>To analyze whether the superiority of Metoprolol Succinate treatment is kept up to three months after the last drug intake. To quantitatively describe and compare the median duration and severity of vertigo attacks, the number of head-

	ache days, neurological and neuro-orthoptic examination and the change of handicap / impairment due to vertigo or dizziness vs. placebo. To check for the occurrence of the adverse effects reported in the summary of the medical product characteristics (SmPC) of the drug.
**Trial Endpoints**	Primary Endpoint:<br>• The number of vertigo attacks and the number of headache attacks during the last 3 months of the 6-month treatment period.<br>Secondary Endpoints:<br>• The number of vertigo attacks and the number of headache attacks during the last 3 months of the total follow-up period of 9 months;<br>• the median duration and severity of vertigo attacks during the last 3 months of the 6-month treatment period and the last 3 months of the total follow-up period;<br>• the number of headache days per month during the last 3 months of the 6-month treatment period and the last 3 months of the total follow-up period;<br>• the changes in the neurological and neuro-orthoptic examination and handicap / impairment due to vertigo between baseline, 6-month visit and 9-month visit.
**Subject Number**	To be assessed for eligibility:<br>Total number of 380 patients<br>To be allocated:<br>Total number of 266 patients, i.e. 133 patients per treatment arm.<br>To be analyzed:<br>Total number of 212 patients, i.e. 106 patients per treatment arm.
**Inclusion Criteria**	Subjects will only be included in the study if they meet all of the following criteria:<br>• Patients male or female, aged 18 years or above,<br>• Patients with diagnosis of probable (1., 4., 5.) or definite (1., 2., 3., 5.) vestibular migraine according to the criteria of Neuhauser (Neuhauser et al. 2001):<br>1. episodic vestibular symptoms of at least moderate severity (rotational vertigo, other illusory self or object motion, positional vertigo, head motion intolerance, i. e., sensation of imbalance or illusory self or object motion that is provoked by head motion);<br>2. migraine according to the IHS criteria;<br>3. at least one of the following migrainous symptoms during at least two vertiginous attacks: migrainous headache, photophobia, phonophobia, visual or other auras;<br>4. at least one of the following: migraine according to the IHS criteria; migrainous symptoms during vertigo as specified in 3.; migraine-specific precipitants of vertigo, e. g. specific foods, sleep irregularities, hormonal changes; response to antimigraine drugs<br>5. other causes ruled out by appropriate investigations;

	• Between 6 and 30 attacks per 3 subsequent months;<br>• Subjects with the ability to follow study instructions and likely to attend and complete all required visits;<br>• Written informed consent of the subject
**Exclusion Criteria**	Subjects will not be included in the study if any of the following criteria applies:<br>• Patients not able to give consent;<br>• Other vestibular disorders such as Menière´s disease, phobic postural vertigo, benign paroxysmal positioning vertigo, vestibular paroxysmia;<br>• Central disorders such as paroxysmal brainstem attacks, TIAs;<br>• Contraindications for the treatment with metoprolol such as<br>o known allergic reaction to the trial drug or other beta receptor blockers;<br>o shock, acidosis<br>o Any bronchospastic disease, e.g. bronchial asthma;<br>o Sick sinus syndrome, known SA-block, AV-block;<br>o Bradycardia < 50 bpm at rest, systolic blood pressure < 100 mmHg, end-grade peripheral arterial disease;<br>o Known severe coronary heart disease or heart failure;<br>o Concurrend treatment with MAO-inhibitors, sympathomimetic drugs, Catecholamine-depleting drugs, digitalis glycosides;<br>• Poorly controlled diabetes mellitus;<br>• Pheochromocytoma;<br>• Suspicion of developing thyrotoxicosis;<br>• Disorders of hemostasis;<br>• Porphyria;<br>• Psoriasis;<br>• Pregnancy or breast-feeding;<br>• Persistent hypertension with systolic blood pressure > 180 mmHg or diastolic BP > 110 mmHg (mean of 3 consecutive arm-cuff readings over 20-30 minutes) that cannot be controlled by antihypertensive therapy;<br>• Life expectancy < 12 months;<br>• Other serious illness, e.g., severe hepatic, cardiac, or renal failure, acute myocardial infarction, neoplasm or a complex disease that may confound treatment assessment;<br>• Treatment with beta-blockers<br>• The patient has received any investigational medication within 30 days prior to administration of study medication or is scheduled to receive an investigational drug up to 30 days after end of study;

	• The patient was previously admitted to this trial or simultaneous participation in another clinical trial or participation in any clinical trial involving an administration of investigational medicinal product.
**Trial Procedures**	**Visit 0** (Screening visit)<br>• Informed consent,<br>• Medical history,<br>• Laboratory (blood sample analysis, unless blood sample analysis not older than 1 week is available and pregnancy testing for women of child-bearing potential),<br>• Check of inclusion and exclusion criteria.<br><br>**Visit 1** (day of inclusion)<br>• Randomization,<br>• Physical examination,<br>• Oculography (incl. caloric testing),<br>• VEMP,<br>• Subjective visual vertical,<br>• DHI Questionnaire,<br>• Dizziness diary,<br>• First administration of trial drug,<br>• Dispensing of trial drug.<br><br>**Visit 2** (1 month after inclusion, study visit)<br>• Oculography,<br>• VEMP,<br>• Subjective visual vertical,<br>• DHI Questionnaire,<br>• Dizziness diary,<br>• Trial Drug / Compliance Check,<br>• Concurrent Medication,<br>• AEs and SAEs.<br><br>**Visit 3, 5, and 6** (2, 4, and 5 months after inclusion, telephone interview),<br>• Dizziness diary,<br>• Trial Drug / Compliance Check,<br>• Concurrent Medication,<br>• AEs and SAEs.<br><br>**Visit 4** (3 months after inclusion, study visit)<br>• Oculography,<br>• VEMP,<br>• Subjective visual vertical,<br>• DHI Questionnaire,<br>• Dizziness diary,<br>• Trial Drug / Compliance Check,<br>• Concurrent Medication,<br>• AEs and SAEs.

	**Visit 7** (6 months after inclusion, study visit)<br>• Oculography,<br>• VEMP,<br>• Subjective visual vertical,<br>• DHI Questionnaire,<br>• Dizziness diary,<br>• Trial Drug / Compliance Check, return,<br>• Concurrent Medication,<br>• AEs and SAEs,<br>• Return of trial drug except for 47.5 mg capsules lasting for 2 weeks.<br><br>**Visit 8** (9 months after inclusion, End of study visit)<br>• Physical examination,<br>• Oculography (incl. caloric testing),<br>• VEMP,<br>• Subjective visual vertical,<br>• DHI Questionnaire,<br>• Dizziness diary,<br>• Concurrent Medication,<br>• AEs and SAEs,<br>• Remaining IMP is returned at Visit 8.
**Trial Specific Measurements**	• Electro- or videooculography<br>• Vestibular Evoked Myogenic Potential Testing (VEMP)<br>• Subjective visual vertical<br>• Dizziness handicap inventory (DHI) Questionnaire<br>• Dizziness diary
**Investigational trial sites**	This is a multi-center trial with 8 investigational trial sites in Germany planned at the time of finalization of the clinical trial protocol.<br>Integrated Center for Research and Treatment of Vertigo,<br>Balance and Ocular Motor Disorders (IFB), University of Munich<br><br>Department of ENT, Technical University of Aachen<br><br>Department of ENT, Technical University of Munich<br><br>Department of ENT, University of Tübingen<br><br>Department of Neurology, Schlosspark-Klinik, Berlin<br><br>Department of Neurology, City Hospital of Celle<br><br>Department of Neurology, University of Essen<br><br>Department of Neurology, Municipal Hospital Altötting-Burghausen
**Statistical Rationale**	Efficacy:<br><br>The two primary efficacy endpoints are the number of vertigo attacks and the number of headache attacks in the two treatment arms during the last 3 months of the 6-month treatment period. As none of the parameters is considered to be normally distributed, non-parametrical testing as in the primary efficacy analysis will be applied.

	Primary statistical Analysis:<br>The statistical analysis of this two-arm study hierarchically tests the two primary endpoints for differences in the above listed order using the Wilcoxon-Mann-Whitney test. The multiple significance level is alpha = 5%. The primary analysis follows the ITT principles.<br>Secondary Endpoints:<br>Mainly, the same non-parametrical tests as in the primary efficacy analysis will be employed.<br>Safety Analysis:<br>Patients will be contacted once a month and be asked about possible side effects in a structured interview, which will be analyzed every month in all two groups. Safety analysis will be performed descriptively at least in 12 months intervals.
**Time Schedule**	Per Patient:<br>• Duration of treatment: 6 month,<br>• Duration of follow-up: 3 month,<br>• Total duration: 9 month.<br>Study duration:<br>• Recruiting Period (first patient in to last patient in): 4 years,<br>• Study duration (first patient in to last patient out): 4 years and 9 month

2 Schedule of Activities

	Screening	Visit 1 (Day of in-clusion)	Visit 2 (1 month after inclu-sion)	Visit 3 (2 months after inclu-sion; tele-phone inter-view)	Visit 4 (3 months after inclu-sion)	Visit 5 (4 months after inclu-sion; tele-phone inter-view)	Visit 6 (5 months after inclu-sion; tele-phone inter-view)	Visit 7 (6 months after inclu-sion)	Final Visit (Visit 8, 9 months after inclu-sion)
Informed Consent	√								
Medical History	√								
Laboratory (Blood sample) [1,2]	√								
ECG	√								
In-/Exclusion Criteria	√								
Randomization		√							
Physical Examination		√							√
Oculography		√	√		√			√	√
VEMP		√	√		√			√	√
Subjective Visual Vertical		√	√		√			√	√
DHI Questionnaire		√	√		√			√	√
Dizziness diary		√	√	√	√	√	√	√	√
Trial Drug / Compliance Check		dispension	√	√	√	√	√	√, return	√, return
Concomittant Medication		√	√	√	√	√	√	√	√
AEs and SAEs			√	√	√	√	√	√	√

[1] incl. negative pregnancy test for women of childbearing potential
[2] Unless blood sample examination not older than 1 week is available

3 Abbreviations

**AE**	Adverse Event
**AMG**	Arzneimittelgesetz
**AR**	Adverse (Drug) Reaction
**BfArM**	Bundesinstitut für Arzneimittel und Medizinprodukte
**BOB**	Bundesoberbehörde
**BW**	Body Weight
**CA**	Competent Authority
**CRA**	Clinical Research Associate
**CRF**	Case Report Form
**CRO**	Contract Research Organization
**CSC**	Clinical Study Center (CSC^{LMU}), Munich
**DHI**	Dizziness Handicap Inventory
**EC**	Ethics Committee
**ECG**	Electrocardiography
**FPFV**	First Patient First Visit
**GCP**	Good Clinical Practice
**ICH**	International Conference on Harmonization
**IHS**	International Headache Society
**IMP**	Investigational Medicinal Product
**ISF**	Investigator Site File
**LKP**	Leiter der klinischen Prüfung
**LPLV**	Last Patient Last Visit
**PEI**	Paul Ehrlich Institute
**SAE**	Serious Adverse Event
**SAR**	Serious Adverse Reaction
**SDP**	Sponsor Delegated Person
**SmPC**	Summary of Medicinal Product Characteristics (“Fachinformation”)
**SUSAR**	Suspected Unexpected Serious Adverse Reaction
**UAR**	Unexpected Adverse Reaction
**VEMP**	Vestibular Evoked Myogenic Potential

5 Introduction

During the last 20 years vestibular migraine has been identified as a distinct type of migraine with the leading symptom of vertigo. There is, however, still an ongoing lively debate on its diagnostic criteria (Neuhauser et al. 2001), (Lempert & Neuhauser 2005), (Neuhauser et al. 2006), (Olesen 2005), (Strupp & Brandt 2008), since vertigo occurs in 30% of the affected patients as a recurrent isolated symptom, i.e., without headache or other migrainous symptoms. Hence for a clinical trial clear diagnostic criteria for migrainous vertigo as given by Neuhauser et al. (Neuhauser et al. 2001) and operationalized capture of symptoms are necessary.

Based on these diagnostic criteria and on validated neuro-otologic interviews (Furman et al. 2003), (Marcus et al. 2004) the prevalence of migrainous vertigo in the general adult population was estimated in the German neuro-otologic survey: its lifetime prevalence was 0.98% and the 12-month prevalence 0.89% (Neuhauser 2007). The similarity of these two numbers suggests that these patients suffer chronically from this condition. In a specialized dizziness clinic vestibular migraine is the most frequent cause of spontaneous recurrent attacks of vertigo and accounts for approximately 10% of the patients (Strupp et al. 2010). The majority of patients with vestibular migraine are middle-aged and in the middle of their working lives.

Attacks typically induce disability and result in considerable economical and social losses. The preventive treatment of migraine attacks is the core approach for patients with frequent headache attacks, as well as in individuals with attack-related disability who are not responsive to acute therapy alone.

5.1 Background

<u>Current clinical knowledge:</u>

There are no large systematic controlled clinical studies for the prophylactic treatment of vestibular migraine, so there is little evidence for an effective medication. Earlier findings were based on case reports or retrospective and observational studies of small groups (Johnson 1998; Reploeg & Goebel 2002; Bisdorff 2004; Carmona & Settecase 2005).

The following drugs have been recommended as prophylactic treatment for vestibular migraine: beta-blockers, valproic acid, lamotrigine (Bisdorff 2004), tricyclic antidepressants and topiramate (Carmona & Settecase 2005). In an observational study on 81 patients the effects of tricyclic antidepressants, beta-blockers, or calcium-channel blockers in combination with diet were evaluated. Seventy-two percent of the patients showed a good response (Reploeg & Goebel 2002). A controlled study on the efficacy of oral triptans (zolmitriptan) during an acute attack of vestibular migraine was conducted in less than 20 patients and remained inconclusive due to its limited power(Neuhauser et al. 2003).

Since the clinical examination of patients with migraine are usually normal between attacks, extending our knowledge on persistent abnormalities would forward diagnostic possibilities. Activation in the brain stem during migraine attacks and permanent cerebellar signs in common forms of migraine have been reported. Both structures are involved in the generation of eye movements and vertigo. Indeed, pathological changes in smooth pursuit eye movements were found in a study of 25 migraine patients(Wieser et al. 2004).

<u>Trial drug:</u>

The trial drug metoprolol is listed as group 1 medication (drugs of first choice) with proven efficacy in migraine prevention (Evers 2008; Diener et al. 2008) and is commonly used in the treatment of vestibular migraine. So far its indication in vestibular migraine is only based on expert opinion as placebo-controlled trials are pending.

Metoprolol was generally well tolerated and the number of reported side-effects was similar to those reported during placebo intake (Olsson u. a. 1984).

Dose Rationale:

In previous studies for the prophylactic use of metropolol in migraine patients, the drug was given in a dosage of 200 mg once daily or 50 mg twice daily. Both treatments reduced the average frequency of migraine significantly and were well tolerated (Wörz et al. 1992). In the European Federation of Neurological Societies 2009 guideline metoprolol was recommended as drug of first choice with a daily dose of 50 – 200 mg given orally (Evers et al. 2009).

As patients included in the study had no cardiovascular indication for the therapy with beta-blockers, the reduction of metoprolol can be performed as described under 10.2.3.

5.2 Trial Rationale

Despite the high prevalence, high burden of disease, and major socioeconomic impact of vestibular migraine, there are no controlled, double-blind studies on its therapy. Beta-blockers, valproic acid, lamotrigine (Bisdorff 2004), tricyclic antidepressants and topiramate (Carmona & Settecase 2005) have been recommended as prophylactic therapy. In an observational study on 81 patients, 72% of the patients showed good responses to tricyclic antidepressants, beta-blockers, or calcium-channel blockers in combination with a diet (Reploeg & Goebel 2002). A controlled study on the efficacy of oral triptans (zolmitriptan) was conducted in less than 20 patients and remained inconclusive due to its limited power (Neuhauser et al. 2003).

In conclusion, a multi-center approach with a controlled, double-masked design is imperative for studies on the prophylactic treatment of vestibular migraine in which the differential effects on vertigo and / or headache should also be evaluated. In such a trial the diagnostic criteria should be very strict and conservative power estimation has to be taken into consideration.

This is a placebo-controlled study. The treatment group (metoprolol succinate 95 mg once daily) will be compared with the group receiving placebo. The placebo group is justified by the lack of sufficient trials proving or disproving an effect of any medication in the treatment of vestibular migraine because there are no adequate placebo-controlled trials for any drug in the therapy for vestibular migraine at all. Regardless of the treatment group patients are allowed to take acute therapy and benefit by the close monitoring, resulting in a precise documentation of their symptoms over a period of time. Even in the treatment of non-vestibular migraine, where effects of prophylactic medication were shown, it is no first-line therapy: its prescription is often delayed, until the patient returns after several weeks or months having completed a patient diary. We did not include flunarizine, valproic acid, or topiramate, although they are licensed for the treatment of migraine with (and without) aura due to their side effects that restrict their use. Flunarizine, for instance, often triggers depressive episodes, and it is well known that patients suffering from vestibular migraine show a higher psychiatric comorbidity than patients with other vertigo syndromes (Best et al. 2006; Warninghoff et al. 2009). Further, flunarizine can cause severe, persistent hypokinetic-rigid syndromes in elderly patients, and about 30% of the patients with vestibular migraine have a symptom onset in their fifth decade of life (Dieterich & Brandt 1999). Finally, valproic acid and topiramate are less well tolerated than metoprolol and several contraindications would considerably limit the number of patients included.

5.3 Side effects and Risk Benefit Assessment

5.3.1 Side effects

Very frequent (> 10%) side effects:

- Tiredness and dizziness
- Headache

Frequent (1-10%) side effects:
- Depression
- Shortness of breath, exertional dyspnea
- State of exhaustion
- Bradycardia
- Diarrhea
- Pruritus, dermal redness and rash
- Cold extremities
- Paresthesia
- Insomnia and nightmares
- Arterial insufficiency, usually of the Raynaud type
- Palpitations
- Congestive heart failure
- Peripheral edema
- Hypotension and orthostatic hypotension, sometimes with syncopes
- Wheezing (bronchospasm) and dyspnea
- Nausea, gastric pain, constipation, flatulence, and heartburn

Occasionally (0.1 – 1%) side effects:
- Difficulties in concentrating
- Diaphoresis
- Muscular cramps
- Vomitting

Rare side effects (0.01 – 0.1%):
- Gangrene in patients with pre-existing severe peripheral circulatory disorders
- Reversible alopecia
- Agranulocytosis
- Dry eyes, conjunctivitis, blurred vision
- AV block first degree and arrhythmia
- Further depression of a pre-existing myocardial contractility
- Nervousness and anxiousness
- Hypoglycemia after extended strong fasting or powerful physical exercises
- Dry mouth, rhinitis
- Erectile dysfunction and loss of libido

Very rare side effects (< 0.01%):
- Hepatitis, jaundice and non-specific hepatic dysfunction
- Photosensitivity and worsening of psoriasis
- Weight gain, arthritis, and retroperitoneal fibrosis
- Peyronie's disease in fewer than 1 of 100,000 patients
- Isolated cases of transaminase, alkaline phosphatase, and lactic dehydrogenase elevations
- Thrombocytopenia and leucopenia
- Mental confusion, hallucination, short-term memory loss and personality change
- Musculoskeletal pain and weakness, arthralgia
- Tinnitus, defective hearing and dysgeusia
- Precordial pain, worsening of attacks in pre-existing angina pectoris, cardiac arrest
- Reduction of renal function in pre-existing severe renal dysfunction

WARNINGS

Beta blockers can cause an increase of sensibility to allergens and of the severeness of anaphylactic reactions.

Beta blockers can cause a dysfunction of the lipometabolism. A reduction of the high-densitiy lipoprotein combined with an elevation of the triglycerides and normal total cholesterol was found.

Currently, not enough experiences exist with Metoprolol Succinate in patients suffering from congestive heart failures with the following concomitants:
- instable heart failure, NYHA Class IV (patients with hypoperfusion, hypotension and/or pulmonary edema),
- acute myocardial infarction or instable angina pectoris during the last 28 days,
- patients over the age of 80 years or under the age of 40 years,
- hemodynamic relevant cardiac valve dysfunction,
- hyperthrophic obstructive cardiomyopathy.

Treatment with Metoprolol Succinate can lead to positive results in doping tests.

In Patients Without a History of Cardiac Failure: Continued depression of the myocardium with beta-blocking agents over a period of time can, in some cases, lead to cardiac failure. At the first sign or symptom of impending cardiac failure, patients should be fully digitalized and/or given a diuretic. The response should be observed closely. If cardiac failure continues, despite adequate digitalization and diuretic therapy, Metoprolol Succinate should be withdrawn.

Major Surgery: The necessity or desirability of withdrawing beta-blocking therapy prior to major surgery is controversial; the impaired ability of the heart to respond to reflex adrenergic stimuli may augment the risks of general anesthesia and surgical procedures. Beta blockers are competitive inhibitors of beta-receptor agonists, and their effects can be reversed by administration of such agents, e.g., dobutamine or isoproterenol. However, such patients may be subject to protracted severe hypotension. Difficulty in restarting and maintaining the heart beat has also been reported with beta blockers.

Bradycardia: Metoprolol Succinate produces a decrease in sinus heart rate in most patients; this decrease is greatest among patients with high initial heart rates and least among patients with low initial heart rates. Acute myocardial infarction (particularly inferior infarction) may in itself produce significant lowering of the sinus rate. If the sinus rate decreases to 50-55 beats/min, the dose should be reduced step by step or the treatment should be stopped respectively. If the sinus rate decreases to < 40 beats/min, particularly if associated with evidence of lowered cardiac output, atropine (0.25-0.5 mg) should be administered intravenously. If treatment with atropine is not successful, Metoprolol Succinate should be discontinued and cautious administration of isoproterenol or installation of a cardiac pacemaker should be considered.

Hypotension: If hypotension (systolic blood pressure ≤ 90 mmHg) occurs, Metoprolol Succinate should be discontinued, and the hemodynamic status of the patient and the extent of myocardial damage carefully assessed. Invasive monitoring of central venous, pulmonary capillary wedge, an arterial pressures may be required. Appropriate therapy with fluids, positive inotropic agents, balloon counterpulsation, or other treatment modalities should be instituted. If hypotension is associated with sinus bradycardia or AV block, treatment should be directed at reversing these (see above).

Diabetes and Hypoglycemia: Beta blockers may mask tachycardia occurring with hypoglycemia, but other manifestations such as dizziness and sweating may not be significantly affected. In rare cases a latent diabetes can become manifest or an pre-existing diabetes can be worsened.

Metoprolol Succinate should not be given in patients with the rare hereditary galactose intolerance, decreased lactase activity or glucose-galactose malabsorption.

PRECAUTIONS

Information for Patients: Patients should be advised to take Metoprolol Succinate regularly and continuously, as directed, with or immediately following meals. If a dose should be missed, the patient should take only the next scheduled dose (without doubling it). Patients should not discontinue Metoprolol Succinate without consulting the physician. Patients should be advised (1) to avoid operating automobiles and machinery or engaging in other tasks requiring alertness until the patient's response to therapy with Metoprolol Succinate has been determined; (2) to contact the physician if any difficulty in breathing occurs; (3) to inform the physician or dentist before any type of surgery that he or she is taking Metoprolol Succinate.

CYP2D6 Inhibitors: Potent inhibitors of the CYP2D6 enzyme may increase the plasma concentration of Metoprolol Succinate. Strong inhibition of CYP2D6 would mimic the pharmacokinetics of CYP2D6 poor metabolizer. Caution should therefore be exercised when coadministering potent CYP2D6 inhibitors with Metoprolol Succinate. Known clinically significant potent inhibitors of CYP2D6 are antidepressants such as fluoxetine, paroxetine or bupropion, antipsychotics such as thioridazine, antiarrhythmics such as quinidine or propafenone, antiretrovirals such as ritonavir, antihistamines such as diphenhydramine, antimalarials such as hydroxychloroquine or quinidine, antifungals such as terbinafine and medications for stomach ulcers such as cimetidine.

Pediatric Use: Safety and effectiveness in pediatric patients have not been established.

From the Summary of Product Characteristics of the following German products: Beloc-Zok®, Prelis®, Lopresor®, Metoprolol-CT 50 mg/100mg/200mg Tabletten, Metoprolol STADA®.

5.3.2 Risk Benefit Assessment

As outlined above Metoprolol Succinate can be expected to reduce the frequency and severity of attacks.

It is a known drug used for many years in common diagnoses such as hypertension and migraine. The drug is generally well tolerated and the number of reported side-effects was similar to those reported in comparison the placebo intake in several studies listed above.

All patients regardless of whether they belong to the verum or placebo group undergo the same non-invasive examinations such as electro- or videooculography, electronystagmography, vestibular evoked myogenic potential testing, subjective visual vertical, and fill in questionnaires as described under 10. Routine blood-samples are taken to exclude liver or kidney failure and pregnancy, if applicable. These precautions are undertaken to provide the patients safety.

Patients are seen on a regular basis to detect potentially unknown side effects. Furthermore, the medical care provided during the clinical trial exceeds the level of standard care.
In summary, it is reasonable to assume equipoise on the basis of possible risks and disadvantages compared to the potential gain in reduction of frequency and severity of attacks.

6 Trial Objectives and Endpoints

6.1 Primary Objective

To demonstrate the superiority of Metoprolol Succinate treatment regarding the number of vertigo attacks and the number of headache attacks per month compared to placebo.

6.2 Primary Endpoint

Primary efficacy endpoints are the number of vertigo attacks and the number of headache attacks per month during the last 3 months of the 6-month treatment period. Frequency of vertigo attacks as well as the frequency of headache attacks will be documented by the subjects by means of a standardized dizziness and vertigo diary.

6.3 Secondary Objective

To analyze whether the superiority of Metoprolol Succinate treatment is kept up to three months after the last drug intake compared to placebo. To quantitatively describe and compare the median duration and severity of vertigo attacks, the number of headache days, the change in the neurological and neuro-orthoptic examination and the change of handicap / impairment due to vertigo or dizziness vs. placebo. To check for the occurrence of the adverse effects reported in the summary of the medical product characteristics (SmPC) of the drug.

6.4 Secondary Endpoints

The duration and severity of vertigo attacks as well as the number of headache days during the last three months of the treatment period and during the last three months of the total follow-up period; the number of vertigo attacks and the number of headache attacks per month during the last three months of the total follow-up period; neurological (smooth pursuit eye movements, Romberg test) and neuro-orthoptic examination (head-shaking nystagmus, subjective visual vertical); change of handicap / impairment due to vertigo or dizziness between baseline, 6-month, and 9-month follow-up visits, assessed by the Dizziness Handicap Inventory (DHI).

Occurrence of new / severe vertigo or dizziness, as well as any of the adverse effects reported in the summary of medical product characteristics (SmPC) on each drug.

7 Trial Design

7.1 Trial Design

- Phase III
- Efficacy of treatment trial
- Multicenter trial
- Prospective trial
- Placebo-controlled
- Randomized
- Double blind
- Parallel, two groups
- Fixed sample

Trial Flowchart:

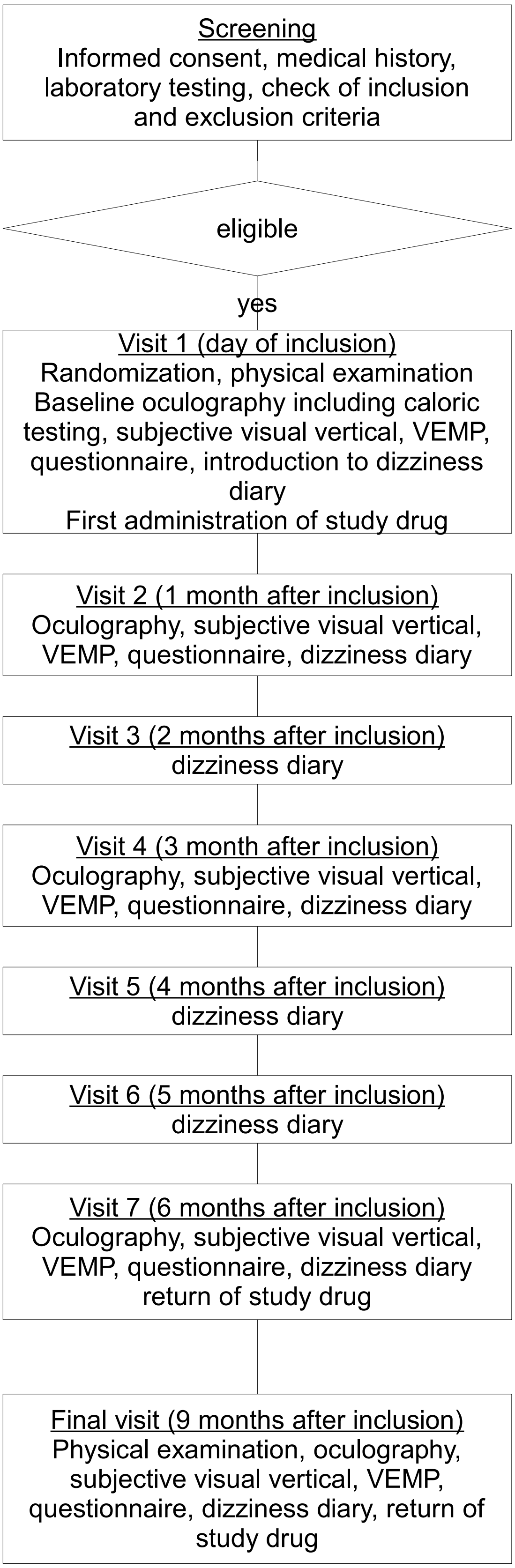

7.2 Number of subjects

In this trial a total of 266 subjects will be included, who will be randomized to either the verum or the placebo group (133 per group).

7.3 Time Schedule

Per Patient:

- Duration of treatment: 6 month,
- Duration of follow-up: 3 month,
- Total duration: 9 month.

Study duration:

- Recruiting Period (first patient in to last patient in): 4 years,
- Study duration (first patient in to last patient out): 4 years and 9 month

The end of the clinical trial is defined by the last individual trial-specific examination during the last visit of the last subject.

8 Trial Population and Selection Criteria

This trial can fulfill its objectives only if appropriate subjects are enrolled. The following eligibility criteria are designed to select subjects for whom protocol treatment is considered appropriate. All relevant medical and non-medical conditions should be taken into consideration when deciding whether this protocol is suitable for a particular subject.

8.1 Gender Distribution

No gender ratio has been stipulated in this trial, as the results of preclinical and / or clinical studies or medical literature did not indicate any difference in the effect of the trial treatment in terms of efficacy and safety.

8.2 Inclusion Criteria

Subjects must meet all of the following inclusion criteria to be eligible for enrolment into the trial:

- Patients male or female, aged 18 and above;
- Patients with diagnosis of probable (1., 4., 5.) definite (1., 2., 3., 5.) vestibular migraine according to the criteria of Neuhauser et al. 2001:
    1. episodic vestibular symptoms of at least moderate severity (rotational vertigo, other illusory self or object motion, positional vertigo, head motion intolerance, i. e. sensation of imbalance or illusory self or object motion that is provoked by head motion);
    2. migraine according to the IHS criteria;
    3. at least one of the following migrainous symptoms during at least two vertiginous attacks: migrainous headache, photophobia, phonophobia, visual or other auras;
    4. at least one of the following: migraine according to the IHS criteria; migrainous symptoms during vertigo as specified in 3.; migraine-specific precipitants of vertigo, e. g. specific foods, sleep irregularities, hormonal changes; response to antimigraine drugs
    5. other causes ruled out by appropriate investigations;

- Between 6 and 30 attacks per 3 subsequent months;
- Subjects with the ability to follow study instructions and likely to attend and complete all required visits;
- Written informed consent of the subject.

8.3 Exclusion Criteria

Subjects will not be included in the study if any of the following criteria applies:

- Patients not able to give consent;
- Other vestibular disorders such as Menière´s disease, phobic postural vertigo, benign paroxysmal positioning vertigo, vestibular paroxysmia;
- Central disorders such as paroxysmal brainstem attacks, TIAs;
- Contraindications for the treatment with metoprolol such as
  - known allergic reaction to the trial drug or other beta receptor blockers;
  - shock, acidosis
  - Any bronchospastic disease, e.g. bronchial asthma;
  - Sick sinus syndrome, known SA-block, AV-block;
  - Bradycardia < 50 bpm at rest, systolic blood pressure < 100 mmHg, end-grade peripheral arterial disease;
  - Known severe coronary heart disease or heart failure;
  - Concurrend treatment with MAO-inhibitors, sympathomimetic drugs, Catecholamine-depleting drugs, digitalis glycosides;
- Poorly controlled diabetes mellitus;
- Pheochromocytoma;
- Suspicion of developing thyrotoxicosis;
- Disorders of hemostasis;
- Porphyria;
- Psoriasis;
- Pregnancy or breast-feeding;
- Persistent hypertension with systolic blood pressure > 180 mmHg or diastolic BP > 110 mmHg (mean of 3 consecutive arm-cuff readings over 20-30 minutes) that cannot be controlled by antihypertensive therapy;
- Life expectancy < 12 months;
- Other serious illness, e.g., severe hepatic, cardiac, or renal failure, acute myocardial infarction, neoplasm or a complex disease that may confound treatment assessment;
- Treatment with beta-blockers
- The patient has received any investigational medication within 30 days prior to administration of study medication or is scheduled to receive an investigational drug up to 30 days after end of study;

- The patient was previously admitted to this trial or simultaneous participation in another clinical trial or participation in any clinical trial involving an administration of investigational medicinal product.

8.4 Subject Information and Recruitment

Patients will be invited to participate by the Principal Investigator responsible for the site or delegated medical doctors. A full verbal explanation of the trial and the Patient Information will be provided for the patient to consider. This will include detailed information about the rationale, design and personal implications of the trial. Following information provision, patients will have sufficient time to consider participation and the opportunity will be given to discuss the trial with their family and healthcare professionals before they are asked whether they would be willing to take part in the trial.

Assenting patients will then be formally assessed for eligibility and invited to provide written informed consent. Formal assessment of eligibility and informed consent discussion will be undertaken by the Principal Investigator at each individual center or by appropriate medical doctors who are authorized to do so by the Principal Investigator responsible for that site. The consent process should be documented in the patient's medical record. Written informed consent for entry into the trial must be obtained prior to randomization.

8.5 Randomization

This trial is designed as a double-blind trial. Randomization will be performed by the IBE and neither the investigators nor the patients will be informed about the treatment arm to which a patient is allocated. Neither can get access to the randomization list.

Concealed randomization to both treatment arms in the ratio of 1:1 will be performed by the IBE of the University of Munich. The randomization technique is based on randomized balanced blocks with random block length. The procedure considers stratification by the center. According to a pre-specified randomization list the trial kits - consisting of identically appearing boxes containing equal amounts of either placebo or standard-dose metoprolol succinate in randomized sequence - will be signed out with (consecutive) identification numbers. A sealed envelope containing the respective treatment group for unblinding in case of an emergency will be attached. Each trial center receives a pool of trial kits. The identification numbers for the kits stored at each center will be registered at the IBE. The IBE will provide an internet-based randomization tool ("Randoulette"), which chooses one of the trial kits stored at the respective center when a new patient fulfills the inclusion criteria and has signed the informed consent. In this way an immediate registration and randomization of each new subject is guaranteed. The coordinating investigator can thus provide an unblinding of the treatment group for a single patient at any time. Furthermore the amount of trial kits stored at the centers can be checked continuously, and redistribution can be arranged if centers enroll different numbers of patients.

9 Investigational Medicinal Product (IMP)

9.1 Specification of IMP

- Drug name: BelocZok mite®
- Name of manufacturer: AstraZeneca GmbH
- Substance name: Metoprolol Succinate
- Amount of active agent per unit: 47.5 and 95 mg
- Other ingredients: Siliciumdioxide, microcrystalline Cellulose, Ethylcellulose, Hyprolose, Hypromellose, sodium stearyl fumarate (Ph. Eur.), Macrogol 6000, hard paraffine, titanium dioxide

- Pharmaceutical form: Sustained-release tablets, encapsulated in hard gelatine capsules for blinding purposes:
  - The drug will be encapsulated using mannitol and aerosil as filling material. It will be refilled from original packaging into capsules under clean room conditions, primary packaging in blisters and relabeled. All procedures will be performed by the Pharmacy of the Hospital of the University of Heidelberg.
  - Placebo will be an identically appearing capsule filled with mannitol and aerosil. Placebo capsules will be packaged in identical blisters as metoprolol succinate. Manufacturing and packaging will be performed by the Pharmacy of the Hospital of the University of Heidelberg.
- Package: primary packaging: blister with 7 (47.5mg) or 10 (95mg) capsules per unit, PVC/PVDC blisterfoil, transparent-orange for adequate UV and vapour barrier, sealed to aluminium blisterfoil, secondary packaging: folding box, white cardboard, labeled and sealed
- Storage conditions: Do not store > 25°C.

  Instructions for delivery: Unchewed encapsulated sustainded release tablets should be taken with sufficient liquid after meals.

9.2 Packaging and Labeling of IMP

The blisters (primary packaging) with the IMP will be labeled as follows (according to German requirements GCP-V §5(4)):

- Name and address of sponsor
- Name of the drug (it will be mentioned that the blister contains either placebo or verum), pharmaceutical formula, dose
- Charge identification (Ch.-B.)
- Continuous identification number (corresponds to patient randomization number)
- Code of the trial protocol
- Trial short title
- EudraCT No.
- Date of expiry

The folding boxes (secondary packaging) with the blistered IMP will be labeled as follows (according to German requirements GCP-V §5):

- Name, address and telephone number of sponsor
- Name, address and telephone number of manufacturing / packaging pharmacy
- Name of the drug (it will be mentioned that the bottle contains either placebo or verum), pharmaceutical formula, dose, amount, mode of application, dosage
- batch identification (Ch.-B.)
- Continuous identification number (corresponds to patient randomization number)
- Code of the trial protocol

- Trial short title
- EudraCT No.
- Date of expiry
- Instructions for storage: nicht > 25 ° C lagern
- Note: "Arzneimittel zur klinischen Prüfung bestimmt"
- Note: "Prüfpräparat unzugänglich für Kinder aufbewahren"
- Note for destruction: -

9.3 Storage requirements

The investigator will be responsible for ensuring sufficient stocks and correct storage of the IMP(s).

The following storage conditions have to be kept for the IMP(s):

• Metoprolol Succinate and Placebo: Do not store > 25°C

Temperature has to be recorded continuously using a Min-Max-Thermometer. These recordings have to be controlled weekly, the results have to be documented in a temperature log by a Principle Investigator delegated person.

9.4 Handling of IMP at the Site and Drug Accountability

The IMP(s) provided by the Pharmacy of the Hospital of the University of Heidelberg has to be stored in a secure location at the site, preferably in a lockable cabinet with restricted access to the investigator(s) and authorized site staff. Personnel who have access to the trial drug need to be listed (name and responsibilities) on the Authorization and Delegation Log in the Investigator Site File (ISF).

In accordance with all applicable regulation requirements, the investigator is responsible for the IMP / randomized therapy accountability, reconciliation, and record maintenance on appropriate forms. The investigator confirms the complete and intact receipt of the total quantity of the IMP by his / her signature and ensures the correct storage (see also 9.3).

The investigator should ensure that the IMP is only used according to the protocol, and may only dispense the IMP to subjects who have signed the informed consent and who have been enrolled in the trial. The dispensing of the investigational medicinal product to subjects outside of this clinical subject is not permitted.

The monitor will check the complete documentation during the regular monitoring visits and will clarify potential discrepancies between usage and dispensing of the IMP(s). The investigator or the monitor has to inform the sponsor-delegated person or the coordinating investigator in case of deficiency regarding e.g. storage or accountability of the IMP.

Copies of all forms completed at the trial site will be returned to the sponsor-delegated person at the end of the trial and will be collected by monitor during the close-out visit or have to be sent to the sponsor-delegated person on request.

9.5 Dosage, Mode of Application and Dose Schedule

- Dose: 1st week 1 capsule with 47.5 mg per day; after 1st week 95 mg per day; after completion of the treatment 47.5 mg per day for two weeks before stopping using the IMP
- Dose rationale: refer to Section 5.1, Dose Rationale

- Mode of application: oral intake;
- Duration of treatment: 6 months

9.6 Subject Compliance

Patients are asked to take their trial drugs with them for every trial visit, so that by counting the remaining capsules, the compliance can be assessed.

9.7 Return and Disposal of IMP

Date and amount of the IMP dispensed to each subject and returned by subject will be recorded for each subject according to subject / random number in the Drug Accountability Log in the ISF. Therefore the remaining or unused IMP has to be returned to the site by each subject at Visits 7 and 8.

All unused IMP should be destroyed in the clinic pharmacy on behalf of the site. In case of the destruction of the IMP, a destruction form has to be completed with the information about date and location of destruction, sort and amount of the IMP, and contact details of the person who will destroy the IMP.

If destruction on behalf of the site is not possible all unused IMP will be inventoried and packaged (if applicable) for return shipment by the site to the Pharmacy of the University of Heidelberg by the end of the trial. In case of destruction of the IMP, a destruction form has to be completed with the information about date and location of destruction, sort and amount of the IMP, and contact details of the person who will destroy the IMP. In case of shipment, the respective shipping information has to be followed.

Copies of relevant forms completed at the trial site will be returned to the sponsor delegated person at the end of the trial and will be collected by monitor during the close out visit or have to be sent to the sponsor delegated person on request.

9.8 Blinding and Emergency Codes

According to a pre-specified randomization list the trial kits will be signed out with continuous identification numbers, and a sealed envelope containing the respective treatment group for unblinding in case of emergency will be attached. The investigator can thus unblind a single patient at any time.

The identification numbers for the kits stored at each center will be registered at the IBE. The IBE will additionally provide an internet-based randomization tool. In this way the IBE can provide an unblinding of the treatment group for a single patient at any time.

9.9 Unblinding

The trial will be subject and investigator blinded. At the initiation of the trial, the trial site will be instructed on the method for breaking the blind. For emergency cases the procedure is described above (9.8). Blinding codes should only be broken in emergency situations for reasons of subject safety. When the blinding code is broken, the reason must be fully documented and entered on the case report form. Emergency envelopes will be sent back to the sponsor-delegated person and checked for integrity when the respective patient has completed follow-up. (ICH-GCP 5.13.4)

As a matter of principle, unblinding is only performed for patients and investigators after the final statistical analysis. The statistician will be unblinded for grouped statistical analyses regarding efficacy (interim analysis or final analysis) only after the closing of the database (interim database in the case of an interim analysis) following a blinded data review. At interim, the statistician reported results of grouped efficacy analyses only to the DSMB members, and the DSMB members recommend on sharing grouped interim results on efficacy with the sponsor-delegated person / coordinating investigator only if deemed necessary. The DSMB members may also request unblinded data.

Any of the following situations may be reasons for premature unblinding:

- In emergency situations, if it is necessary for the subject's safety, i.e. if the further treatment depends on the knowledge of the investigational medicinal product.
- In the event of accidental administration of the investigational medicinal product to a person who is not a subject.
- In the event of the death of a subject, if a causal relationship between the treatment with the investigational medicinal product and death is suspected.
- In the event of SAEs / SUSARs under certain conditions (causal relationship with the investigational medicinal product).

In emergency situations or in the event of accidental administration of the investigational medicinal product, the decision whether unblinding is necessary lies with the investigator.

9.10 Prior and Concomitant Therapy / Medication

9.10.1 Previous or concomitant therapy / medication

All concomitant therapies / medications other than the trial therapy / investigational medicinal product applied during the trial at the discretion of the investigator will be documented in the subject's medical record and in the appropriate CRF.
The doses of concomitant medications for e.g. chronic diseases should be kept as constant as possible throughout the trial.
Concomitant medication will be recorded during the entire study period.

See also chapter 10.

9.10.2 Prohibited therapy / concomitant medication

The following therapies / medications are not allowed to be applied during the trial:

- any other beta-blockers,
- topiramate,
- valproic acid,
- lamotrigine and / or
- tricyclic antidepressants.

Every effort should be made to avoid the use of any of the listed prohibited therapies / medications during the entire duration of the trial.

If there is a single administration of any of the drugs mentioned above, the subject is still eligible for the main analysis. Regular intake of these drugs is considered a protocol violation. Concomitant medication must be documented.

Otherwise there is no known limitation for concomitant medication. There is no contraindication for the use of any other medication during the treatment period.

9.10.3 Rescue / Escape / Salvage Therapy

Currently, controlled trials for the therapy of vestibular migraine are lacking. In accordance with the guidelines issued by the German Society of Neurology (Diener & Putzki 2008), vestibular migraine attacks can be treated like attacks of migraine with aura, i. e. using non opiod analgesics, non steroidal anti-inflammatory drugs, or triptans.

10 Trial Procedures

If any prophylactic treatment of migraine is already taken by the patient, a wash-out period of at least one month is necessary before starting the trial.

10.1 Methods of Assessment

The following section will give an overview and explanations to the examinations and procedures to be performed in this trial.

Source documents must be stored and be available for subsequent review. The respective printouts will be stored in the subject medical file, including the trial number.

10.1.1 **Oculography (Electro- or Videooculography)**

Depending on the equipment of the local trial centers, eye movements will be recorded using electro- (EOG) or videooculography (VOG).
VOG testing is a series of tests designed to document a person's ability to follow visual objects with their eyes and how well the eyes respond to information from the vestibular system. It allows a real time video and graphical visualization of horizontal, vertical and torsional eye movements without applying electrodes. VOG measures the movements of the eyes directly through infrared cameras, instead of measuring the mastoid muscles around the eyes with electrodes like the previous EOG version. VOG testing is more accurate, more consistent, and more comfortable for the patient. When the patient is more comfortable and relaxed, con-sis-tent and accurate test results are more easily obtained.
VOG testing is used to determine if a vestibular (inner ear) disease may be causing a balance or dizziness problem. To monitor the movements of the eyes, infrared goggles are placed around the eyes to record eye movements during testing in an upright sitting position. VOG and EOG testings are non-invasive, and only minor discomfort is felt by the patients during testing as a result of wearing goggles. Appointments usually last about 1 hour.

10.1.2 **Vestibular Evoked Myogenic Potential Testing (VEMP)**

The VEMP is used to test the reflex arc of the saccule, which extends over the vestibular nerves, vestibular nuclei, interneurons, and motor neurons to the neck musculature (sternocleidomastoid m.). It complements caloric testing, since the letter tests only the canal system and not the otolith function. The prerequisite for VEMP testing is an intact middle ear function; it is not necessary that hearing be preserved, since the "sensitivity to sound" of the saccule can be used in the VEMP. The reflex is triggered by a loud click. Surface EMG is used to record from both sternocleidomastoid muscles.
Healthy subjects first show on the ispsilateral side a positive wave (about 14s after the stimulus) as well as a negative wave (about 21 ms). As a rule the responses cannot be recorded contralaterally. Approximately 50-100 times averaging is necessary for the recording. It is important that the musculature is tense; for this the patient can raise his head from the support surface. Evaluation criteria are the presence of the waves P14 and N21 as well as their amplitude. Both their absence and a clear reduction in amplitude are considered pathological; the relevance of changes in latency must still be determined.

Modified from "Vertigo and dizziness", Brandt Th, Dieterich M, Strupp M, Springer 2009.

10.1.3 **Subjective visual vertical (SVV)**

For determination of the SVV, the patient sits in an upright position with his or her chin resting on a fixed pad looking into a hemispheric dome of 60 cm in diameter. The surface of the dome extends beyond the limits of

the patient's visual field and is covered with a random pattern of colored dots. This prevents the patient from orientating himself spatially by fixed external structures. The hemispheric dome is connected axially to a motor, and can be rotated. A circular target disc (14° of visual field) with a straight line through the centre is placed 30 cm in front of the patient at eye level. The line is also connected with a DC motor. After target and dome are rotated to a randomized offset position, the patient is instructed to align the target with the perceived vertical by using a joystick-device (potentiometer). The deviation of the line from the objective vertical axis is measured in degrees and registered on a PC. The mean of ten measurements equals the SVV. Under these conditions, the normal range (mean ± 2 SDs) of the SVV is 0° ± 2.5°.

Modified from "Vertigo and dizziness", Brandt Th, Dieterich M, Strupp M, Springer 2009.

10.1.4 **Dizziness handicap inventory (DHI) Questionnaire**

To assess the impact of impairment the patients are asked to fill out the 25 item DHI questionnaire, which has been used in previous studies including patients with vestibular migraine. A copy can be found in the .

10.1.5 **Dizziness diary**

Patients will be asked about the frequency of attacks in all telephone interviews and the diaries will be checked in all study visits. The frequency of attacks of vertigo has been used in previous studies on neuro-otological diseases as the standard primary outcome measure, e.g., Menière´s disease, because this is the most disabling symptom (Strupp et al. 2008).

10.1.6 **Laboratory examinations / Biological Specimens**

The following parameters will be determined according to the time schedule given in the flow chart.

A routine blood-sample will be taken to exclude liver or kidney failure, malignant diseases, and pregnancy if applicable.
If a routine blood sample examination not older than one week is available, no new blood testing needs to be done. If applicable, a urine pregnancy test can be performed in this case.
Blood sample examinations older than one week but not older than 30 days are considered a minor protocol violation.

The following measurements are done:

- Sodium (Na),
- Potassium (K),
- Urea,
- Creatinine,
- Glucose,
- Red blood cells (RBC),
- Mean corpuscular volume (MCV),
- Mean corpuscular hemoglobin (MCH),
- Mean corpuscular hemoglobin concentration (MCHC),
- Hemoglobin,
- Hematocrit,
- White blood cells (WBC),
- Platelets,
- Alanine transaminase (ALT),
- Aspartate transaminase (AST),
- Gamma glutamyl transpeptidase (GGT),
- C-reactive protein (CRP) and
- hCG (if applicable).

The total amount of blood drawn per subject during the entire trial will be approximately 8 ml.
In each study center the testing will be done by the in-house laboratory.
The results from the blood test will be reviewed and evaluated by one of the investigators of each single study center.
Clinically significant findings at Visit 0 (Screening visit) which describe the baseline status of the subjects will be documented as concomitant disease under medical history. In case of meeting any exclusion criteria, the patient cannot be included in the study.

10.1.7 **Electrocardiography**

During screening a standard electrocardiograph is recorded in order to rule out cardiac arrhythmia as listed under Exclusion Criteria.

10.2 Time schedule of Measurements

10.2.1 **Screening period**

Visit 0 (Screening visit)

All subjects will be screened for eligibility before enrollment. Only eligible subjects will be enrolled into the trial. Patients will be recruited by the centers' outpatient services and both, examinations and treatment will be performed in an ambulant setting.

All subjects must have the following procedures completed prior to enrollment:

1. Informed consent will be obtained.

2. Medical history and demographic data will be recorded.

3. Inclusion and exclusion criteria will be checked along with the retention of a blood sample and analysis and ECG recording. If applicable, pregnancy testing will be done. The blood sample examination should not be older than one week. Blood sample examinations older than one week but not older than 30 days are considered a minor protocol violation.

4. Female subjects with childbearing potential will be informed about the contraindication of the IMP during pregnancy and breast-feeding and be requested to apply highly effective contraceptive methods during the duration of the trial participation.

10.2.2 **Treatment period**

The treatment period will include 7 visits from day 0 until month 6, so the treatment will be extended to a period of 180 days.
A delay of ± 7 days is acceptable for all visits.
All visits will be performed according to the flow chart (see chapter 7.1).

Visit 1 (Day of inclusion)

- Change of concomitant therapy / medication since last visit,
- Randomization,
- Physical examination,
- Electro- or Videooculography,
- VEMP,
- Subjective visual vertical,

• DHI Questionnaire,
• Dizziness diary,
• Concomittant Medication,
• First administration of trial drug,
• Dispensing of trial drug.

The following assessments and procedures will be performed at **Visits 2 - 7**:

• Dizziness diary,
• Trial Drug / Compliance Check,
• Concomittant Medication,
• AEs and SAEs.

The following assessments and procedures will be performed at **Visits 2, 4 and 7**:

• DHI Questionnaire,
• Electro- or Videooculography,
• VEMP,
• Subjective visual vertical.

The IMP will returned at Visit 7 except for 47.5 mg capsules lasting for 2 weeks (i. e. at least 14 capsules).

10.2.3 Follow-up period

The follow-up period takes place between Visit 7 and Visit 8, and no evaluations will be done. After Visit 7 the patients receive 1 capsule with 47.5 mg verum/placebo per day for two weeks. After these two weeks patients receive no more study medication during the follow-up period.

10.2.4 Final Visit (Visit 8, End of study visit or premature termination)

The following procedures will be performed at the final visit or in subjects who terminate the trial prematurely:

• Physical examination,
• Electro- or Videooculography,
• VEMP,
• Subjective visual vertical,
• DHI Questionnaire,
• Dizziness diary,
• Concurrent Medication,
• AEs and SAEs.
Remaining IMP is returned at Visit 8.

10.2.5 End of the trial

The end of the trial is defined by the last individual trial-specific examination during the last visit of the last subject.

11 Safety Data Documentation and Reporting

11.1 Definitions

Adverse Event (AE)

An Adverse Event / Experience (AE) is any untoward medical occurrence in a patient or in a clinical investigation subject administered a pharmaceutical product and which does not necessarily have a causal relationship with this treatment.

An AE can therefore be any unfavorable and unintended sign (including an abnormal laboratory finding), symptom or disease temporally associated with the use of a medicinal product, whether or not related to the treatment.

Adverse (Drug) Reaction (AR)

In the pre-approval clinical experience with a new medicinal product or its new usages, particularly as the therapeutic dose(s) may not be established: all noxious and unintended responses to a medicinal product related to any dose should be considered as adverse drug reactions. The phrase “responses to a medicinal product” means that a causal relationship between a medicinal product and an adverse event is at least a reasonable possibility, i.e., the relationship cannot be ruled out.

Regarding marketed medicinal products: a response to a drug which is noxious and unintended and which occurs at doses normally used in humans for prophylaxis, diagnosis, or therapy of diseases or for modification of physiological function.

Unexpected Adverse (Drug) Reaction (UAR)

An adverse reaction, the nature or severity of which is not consistent with the applicable product information (e.g., Investigator's Brochure for an unapproved investigational product or package insert / summary of product characteristics for an approved product).

Serious Adverse Event (SAE)

Any untoward medical occurrence that at any dose

• results in death,

• is life-threatening,

• requires inpatient hospitalization or prolongation of existing hospitalization,

• results in persistent or significant disability / incapacity,

• is a congenital anomaly / birth defect,

or

• is qualified as another medically significant event or condition.

Serious Adverse (Drug) Reaction (SAR)

This is defined as an adverse drug reaction that is serious and at least possibly related to the IMP (see SAE criteria above).

Suspected Unexpected Serious Adverse (Drug) Reaction (SUSAR)

A SUSAR is an adverse reaction, which is suspected, serious <u>and</u> unexpected because the nature or severity of this event is not consistent with the applicable product information (e. g., Summary of Product Characteristics for an authorized product or Investigator’s Brochures for an unauthorized investigational medicinal product).

11.2 Criteria to be evaluated by the investigator (1st assessment)

Special attention is to be paid to the occurrence of adverse events (AE) throughout every stage of the clinical trial. The investigator should evaluate all adverse events according to the criteria and steps mentioned below.

11.2.1 **Assessment of Intensity**

Any adverse event has to be graded by its intensity.	
MILD	Does not interfere with subject's usual function, easily tolerated.
MODERATE	Interferes to some extent with subject's usual function.
SEVERE	Interferes significantly with subject's usual function, incapacitating with inability to work or carry out usual activity.

11.2.2 **Assessment of Seriousness**

Determination of the seriousness of the adverse event according to the definitions of a serious adverse event (SAE) given in section 11.1.

11.2.3 **Assessment of Causality**

Determination of the relationship of the adverse events to the medicinal product being studied after having evaluated all accessible data according to the following classification.

Suspected:

The temporal relationship between the event and the administration of the IMP makes a **causal relationship possible, probable, or definite,** or other drugs, therapeutic interventions or underlying conditions do not provide a sufficient explanation for the observed event.

Not suspected:

The temporal relationship between the event and the administration of the IMP makes a **causal relationship unlikely or impossible (i.e., not related),** or other drugs, therapeutic interventions or underlying conditions provide a sufficient explanation for the observed event.

When the final causality assessment is unknown and it is **uncertain** whether or not the investigational product caused the event, then the event should be handled as SAE-**related** (suspected) to the investigational product for reporting purposes.

11.3 Criteria to be evaluated by the Sponsor-Delegated Person (2nd assessment)

In addition to the first evaluation of an adverse event that is performed by the investigator, a second evaluation with respect to seriousness, causality and expectedness and a risk / benefit assessment is performed by the Sponsor-delegated Person to process safety evaluation according to a four eyes principle.

11.4 Documentation and Reporting of Adverse Events

Any AE has to be documented in the CRF on the respective Adverse Event Report Form.

Documentation and evaluation of each AE occurs between

- the visit with the first intervention to the subject and
- the last visit with the last individual specific examination of the subject.

11.5Documentation and Reporting of Serious Adverse Events

Any SAE has to be reported via the SAE Report Form immediately to GKM. GKM will provide this standard form to all investigators.

Documentation and evaluation of each SAE occurs

- after the subject has been randomized and has received the trial drug
- up to 30 days after the subject has received the last dose of the trial drug.

11.5.1 Initial reporting of SAEs

Any SAE has to be documented on the respective Serious Adverse Event Report Form, provided by GKM to all investigators. The investigator has to

Report the SAE to GKM
immediately
after becoming aware of this event
(at the latest within 24 hours)
Fax: +49 (89) 20 91 20 30

Further details for documentation and reporting procedures are described in a SAE Management Plan, provided to all investigators by GKM.

11.5.2 Reporting to the authorities and ethics committees

The **SARs** of a clinical trial have to be notified to the competent authority once a year or on request.

Depending on the sort of the SUSAR the SDP has to consider special timelines for reporting to the competent authorities and ethics committees.

The following timelines have to be considered for reporting of SUSARs to CA and EC:

Fatal or life-threatening SUSARs:

- as soon as possible, at the latest within **7 calendar days** after first knowledge of the minimum criteria

Non-fatal and non-life-threatening SUSARs:

- as soon as possible, at the latest within **15 calendar days** after first knowledge of the minimum criteria.

In multicenter trials the principal investigators of all other investigator sites will be informed about each SUSAR.

All these tasks are delegated to GKM via the CSC by the SDP.

11.6Pregnancy

Women of childbearing potential are required to have a negative pregnancy test to exclude a pregnancy before being enrolled in the clinical trial.

The time period for collecting pregnancy testing information is identical to the time period for collecting AEs as stated in Section 11.4 of the trial protocol.

11.6.1 Actions to be taken if pregnancy occurs to female subjects or partners of male subjects

If a female subject becomes pregnant or is suspected to be pregnant (including a positive pregnancy test regardless of age or disease state) while participating in this trial and taking the study drug, or within 30 days of the last dose of the study drug, the investigator has to be informed immediately about this event.

The pregnant subject has to discontinue the treatment with the IMP permanently, has to be excluded from the trial and has to be instructed to return any unused portion of the study drug to the investigator, if applicable.

Likewise, if the partner of a male trial subject becomes pregnant or is suspected to be pregnant while the subject participates in this trial, the investigator has to be informed immediately by the male subject about this suspected or confirmed pregnancy. The investigator will then provide this information to the sponsor / sponsor-delegated person for follow-up as necessary.

To ensure the safety of female subjects or female partners of male subjects, each pregnancy that becomes known to the investigator during the trial must be reported as an event.

Therefore the investigator will record and report pregnancy information on the appropriate report form as an initial report and fax it immediately (at the latest within 24h) to the sponsor-delegated person.

The pregnancy itself is not considered to be an AE or SAE but must be followed up until delivery or until pregnancy termination and the outcome of pregnancy should be notified to the sponsor-delegated person to determine the outcome of the pregnancy regarding maternal or newborn complications. The investigator will seek and provide this follow-up information after the planned date of delivery. This information will be forwarded to the sponsor-delegated person. For this purpose the pregnancy report form will be used as follow-up report. The timeframe for following-up the details of birth will be no longer than 28 days after the delivery date.

If the outcome of the pregnancy includes

- a spontaneous, therapeutic abortion or voluntary termination,
- stillbirth,
- neonatal death,
- presence of birth defects, or
- congenital anomaly (including that in an aborted fetus, stillbirth or neonatal death),

the investigator should report this outcome as an SAE.

All neonatal deaths that occur within 28 days of birth should be reported as SAEs without regard to causality.

In addition, any infant death after 28 days that the investigator suspects is related to the *in utero* exposure to the study drug should be reported.

Furthermore, any SAE occurring as a result of a post-trial pregnancy **and** considered reasonably related to the investigational medicinal product by the investigator will be reported as described in this Section. The investigator is not obliged to actively seek this information in former trial participants, but has to meet the reporting obligations as soon as the investigator is aware of this event through spontaneous reporting by the person concerned.

12 Data Safety Monitoring Board

An independent Data and Safety Monitoring Board will be installed. The major function of this committee will be to monitor the safety and efficacy of the trial and to provide recommendations regarding further enrollment and conduct of the trial. The DSMB will periodically review tabulated safety summaries and additional safety data which the DSMB may request during the conduct of the trial. The DSMB is responsible for making recommendations to the sponsor regarding modifications or stopping the trial based on safety observations, and, if requested, on efficacy data. Particular attention will be paid to the incidence of particular AEs, including death, flush, severe persisting headache, hypotonia (systolic blood pressure < 100 mmHg), bradycardia (< 50 beats per minute), bronchospasm or Quincke's edema (edema of the upper respiratory tract or the mucosa). The proceedings of the DSMB are specified in the DSMB Manual.

13 Statistic and Analysis

13.1 *Trial Design*

see section 7.1

13.2 Target Variable/Endpoints

see section 6

13.3 Statistical Analyses

Primary efficacy endpoints are the number of vertigo attacks and the number of headache attacks per month during the last 3 months of the 6-months treatment period. Frequency of vertigo attacks as well as the frequency of headache attacks will be documented by the subjects by means of a standardized dizziness and vertigo diary. If not revised in a statistical analysis plan (SAP), at visit 7, the dizziness diaries (from visits 5, 6 and 7) will be evaluated starting with visit 4. The maximal evaluable number of days should be fixed by unambiguously marking those days in the dizziness diary where the investigator suggests insufficient documentation for primary endpoint evaluation. The counted number of attacks relative to the evaluated number of days being in the time interval from 77 days until 196 days after randomisation (and between visit 4, inclusive, and visit 7, exclusive) will define the value of an endpoint for a patient and may be expressed as number of attacks per months. If the number of evaluated days is less than 61 for a patient, a major protocol deviation will be declared. Handling of such protocol deviations will be fixed before unblinding the statistician.

Both primary endpoints will be analysed confirmatory. Aiming to demonstrate superiority of Metoprolol (M) compared to Placebo (P), two-sided tests for detecting differences will be carried out. Multiple testing with strong control of the familywise error rate at a significance level of $\alpha = 5\%$ will be performed. Thereby each of the two primary endpoints will be tested at a type I error level of 5%, however hierarchically, first the number of vertigo attacks followed by the number of headache attacks. Superiority can be claimed based on the num-ber of vertigo attacks alone, Thus the number of headache attacks is regarded as a co-primary endpoint. In case of claimed superiority by demonstrating a reduced frequency of vertigo attacks, the comparison of the frequency of headache attacks is next important.

The two endpoints in the confirmatory statistical analysis are considered to be not normally distributed. Thus, each of the corresponding statistical null hypotheses is the hypothesis that the distribution functions of the respective endpoint in the metoprolol group and the placebo group are equal and will be tested non-parametrically with the Mann-Whitney-Wilcoxon test in order to detect directed differences of the distribu-tions. The distributions will be described by median, minimum, maximum, and quartiles separately for both the placebo and metoprolol groups. If adequate, differences between the groups will be described by use of Hodges-Lehman estimates including confidence intervals. For the purpose of modelling in additional analy-ses using re-

gression techniques, checks for normal distributions after arsinh transformation of the primary endpoints will be conducted.

The primary statistical analysis encompasses the confirmatory closed testing procedure incorporating the two primary endpoints and will be based on the intention to treat (ITT) principle.

Analysis of all secondary endpoints and of patient characteristics is descriptive. Again, due to expected deviations from the normal distribution, descriptive comparisons will be mainly conducted with the Mann-Whitney-Wilcoxon test or, in case of binary outcome, with the Fisher's exact test.

Safety data will be analyzed descriptively in the two groups every 12 months.

13.4 Interim Analysis

A fixed sample design is planned without confirmatory statistical testing for early decision making. However, having documented primary endpoint values of 100 enrolled patients for each primary endpoint in the clinical data base or having reached the time point of 2.5 years after randomization of the first patient (whatever occurs first), an interim analysis is planned in order to check data quality for the primary statistical analysis and assumptions of initial sample size calculation. The interim analysis and interim report will describe pa-tient recruitment, treatment compliance as well as safety and tolerability for the subjects in this period. After data cleaning and analysis the interim report will be submitted to the data monitoring committee (DMSC) to obtain its recommendation.

Efficacy parameters will be analysed. Based on the pooled distribution of the number of (vertigo) attacks per months of the patients at inclusion and the pooled distribution of the number of attacks per months during the first 3 months and during the last 3 month of the 6-months treatment period, a two- or three-group stratifica-tion by the number of (vertigo) attacks at inclusion will be considered for the primary analyses (simplicity versus reduction of variance). In case of substantial differences from the assumptions, the assumptions will be revised and a sample size re-calculation will be performed. At the time of this interim analysis there will be no spending of the type I error level α for testing significance. Nevertheless, efficacy parameters may be analysed on grouped data at any interim analysis or interim look. According to the results of the interim analysis based on grouped data, a redesign (e.g., an adaptation of the sample size) may be considered and communicated with the independent DSMB.

13.5 Modifications of the Statistical Design for Confirmatory Analysis

In order to keep the type I error level in the case of a design modification at any time during the course of the trial, the "Conditional Rejection Probability" approach of Müller and Schäfer *(Müller & Schäfer 2001, 2004)* will be applied. Thereby the Brownian motion approximation with inverse normal transformation of the one-sided p-value at interim with information time $\frac{n_{M,I} \cdot n_{P,I}}{n_{M,I} + n_{P,I} + 1}$ relative to information time $\frac{n_{M,} \cdot n_P}{n_M + n_P + 1}$ at final analysis will be used. In the formulae, $n_{M,I}$ and $n_{P,I}$ denote the numbers of observed values at interim in the respective treatment groups, and n_M=n/2 and n_P=n/2 are the corresponding numbers at final analysis, where n, the total number of observations of an endpoint, is to be planned (see next section). In the case of a modification of the statistical design, the trial protocol has to be amended.

13.6 Sample Size Calculation

Three parameters influence the sample size of the study which uses the Wilcoxon (Mann-Whitney) rank-sum test for the statistical decision: the level of significance, the power of the two-sided test, and the probability that an observation X_M in the metoprolol group is less than an observation X_P in the placebo group. Here, the observations are the values of the primary endpoint expressed in attacks per months based on an observation period of near to or around 3 months. For the primary endpoints a reduction of 1 attack per month due to

metoprolol is regarded as clinically relevant and was set the difference to be detected with the study. The clinical relevance of reductions less than 1 attack per month is debatable. Brandes et al. (Brandes et al. 2004) reported reductions of a little more than 1 attack per month by medication over up to 18 weeks (more than 40% drop off before 18 weeks medication) in migraine prevention where the baseline attack frequency was restricted to 3 to 12 attacks per months. They observed a mean of around 4 attacks per months during medication period with a standard deviation of around 3 attacks per months. In patients suffering from vestibular migraine, the number of vertigo attacks per month rarely exceeds 10. Restricting patient recruitment to patients with vestibular migraine having a history of vertigo attacks of 2 to 10 attacks per months, we expect a standard deviation of the first primary endpoint corresponding to a value of 2 to 2.5 vertigo attacks per month. A **sample size of 106 patients in each group** will have 80% power to detect a probability of 0.611 that an observation X_M is less than an observation X_P using a Wilcoxon (Mann-Whitney) rank-sum test with a 5% two-sided significance level. (The probability of $P(X_M < X_P)=0.611$ was calculated with a presumed normal distribution and an effect size of 1 and a standard deviation of 2.5).

On the basis of our experience with patient compliance in previous studies and routine treatment, we observed a drop-out rate of about 20%. Thus, **a total of n=266 patients (133 in each treatment group)** have to be enrolled. Nevertheless, for the two primary endpoints, we aim to keep the drop-out rate below 10%.

It has to be taken into consideration that about 30% of patients fulfilling the inclusion criteria for this trial might refuse to give their informed consent to participate in the trial; we therefore expect to screen a total of about 380 patients for eligibility.

13.7 Populations included in the Analysis

The primary endpoints of the clinical subject will be analysed primarily according to the intention-to-treat (ITT) principle. Amongst others, this means that, whenever possible, the subjects will be analysed in the treatment arms to which they were randomised, irrespective of whether they refused or discontinued the treatment or whether other protocol violations are revealed.

An analysis per-protocol (PP) for a specific endpoint excluded or censored endpoint information considering major protocol deviations potentially effecting subjects specific endpoint value, e.g. in the case of major violation of eligibility criteria, lack of sufficient treatment by protocol, major use of prohibited concomitant treatment, or unsatisfactory examinations or evaluations for endpoint assessment. Exclusions or censoring with respect to endpoints confirmatory analysed after unblinding of the statistician will be listed and accounted for (e.g. incidentally finding of major violation of eligibility criteria during secondary analyses). The analyses PP group will be performed for the purpose of a sensitivity analysis.

13.8 Protocol Violations

Protocol violations are major deviations from the procedures outlined in this document like:

- missed evaluations
- incorrect timing of evaluations
- non-compliance with investigational medicinal product
- the intake of medications not allowed
- any non-adherence to the protocol that would have an impact to the subject's rights, safety or welfare.

After a subject has been enrolled, it is the investigator's responsibility to make a reasonable effort to correct any protocol violations and to continue the subject's participation in the trial, if possible.

Protocol violations do not constitute a justification for withdrawal of a subject from the trial themselves.

Protocol violations will be reported to the sponsor/sponsor delegated person during the course of the trial in the monitoring reports.

All protocol violations will be listed and the impact on the evaluation of the subjects concerned will be discussed at regular intervals (schedule of intervals depending on the amount of protocol violations) and prior to unblinding for a statistical analysis. Those regarded as major deviations with impact on a confirmatory statistical analysis will be coded with respect to handling in the respective statistical analysis.

13.8.1 **Handling of Drop-outs, Withdrawal, and Missing Data**

It is intended to keep the drop-out proportion rate below 10% for the primary endpoints. Drop-outs will not be replaced.

In the primary statistical analysis, missing values will not be replaced. If not revised in a SAP, values of the primary endpoints will be used in the ITT analysis or PP analysis if they are based on at least 31 or 61 evaluated days, respectively. (See also Sensitivity analyses.)

After the primary ITT analysis, sensitivity analyses will follow per protocol or as treated principles; the latter will also incorporate drug compliance.

Moreover, “pattern-mixture models“ may be applied to handle possible non-ignorable missing data (Little & Wang 1996, Chapter 8 in Fairclough 2002). and the overall effect of treatment on each of the two primary endpoints will be analyzed separately in a longitudinal approach based on a linear mixed effects model.

14 Data Collection, Handling and Record Keeping

14.1 Data Management

Details on data management (procedures, responsibilities, data corrections, if any, which may be made by Data Management staff themselves, etc.) will be described in a **data management plan** prior to the trial. During the trial, the performance of data management and any deviations from the data management plan will be documented in a data management report. Before any data entry is performed, the trial database will be validated and the technical specifications of the database will be documented in a variable-plan.

For hard copies of CRFs are used, double data entry will be performed by two different persons (with the exception of free text). The two entries will then be compared with each other and verified. An audit trail will be created to provide an electronic record of which data were entered or subsequently changed, by whom and when.

SAS software (SAS Institute, Cary, USA) will be used to review the data for completeness, consistency and plausibility. The checks to be programmed will be specified beforehand in a **data validation plan**, as required by the subject protocol. After running the check programs, the resulting queries will be sent to the investigator for review of his/her data. Answered queries will also be entered twice, verified and the updated data will then be transferred to the database. All programs which can be used to influence the data or the data quality will be validated (e.g. check programs, programs used for the input of external data, etc.).

14.2 Data Coding

Coding of adverse events, concomitant diseases and medications will be done according to the following coding systems:

— *MedDRA*

— ATC / WHO-DD

14.3 Documentation of Trial Data

14.3.1 Documentation of Trial Data in the Medical Record

The investigator will record the participation in the trial, the frequency of the trial visits, the relevant medical data, the concomitant treatment and the occurrence of adverse events in the medical record of each subject.

Data collected on the CRFs must match the sources data. These may include but are not limited to the hospitals' or the physician's medical files, laboratory and pharmacy records, diaries etc.

In some cases, the CRF, or part of the CRF, may also serve as source document. In these cases, a document should be available at the investigator's site that clearly identifies those data that will be recorded in the CRF, and for which the CRF will stand as the source document.

14.3.2 Case Report Form (CRF)

The investigator has ultimate responsibility for the accuracy, authenticity, timely collection and reporting of all clinical, safety, laboratory data entered on the CRFs. All these data may only be entered into the CRF by authorized trial personnel as promptly as possible.

Patient diaries and questionnaires (see Chapter 10) will be collected at the designated visits and data will be extracted by the data management. The original diaries and questionnaires will then be archived like other source documents.

14.4 Investigator Site File

The trial site will be provided with an investigator site file (ISF) containing all sponsor-specific essential and trial specific documents. The monitor will regularly check the trial site file for accuracy and completeness. The trial site file has to be stored locked and secure. After end of trial or early termination of the trial the trial site file should be retained for 10 years at the site.

The ISF includes the subject identification list, where the investigator has to record the trial participation of each subject. This list allows identification of each subject and contains the subject number, the name, telephone number (if applicable), birth date and the date of inclusion of the subject into the trial, and will be reviewed by the monitor for completeness. After end of the trial the subject identification list remains with the subject site. In addition, trial participation of the subject should be recorded in the subject chart (trial drug, screening / randomization number, start and end date of the trial).

The investigator should maintain a list of appropriately qualified persons to whom he or she has delegated trial duties. This list will be provided with the ISF, too.

Furthermore, trial personnel responsible for documentation in the CRFs should be identifiable. Therefore a signature list with the name, signature, initials / abbreviations and trial responsibilities of all persons who are allowed to make entries into the CRF will be filed in the investigator's site file.

The trial documents provided by the sponsor / CSC are confidential and may not be made accessible to third parties not involved in the trial by the investigator or other staff members. All trial data are collected pseudonymously.

14.5 Archiving

14.3.1 Sponsor

The sponsor must archive all trial related documents according to regulatory requirements.

14.5.1 **Investigator**

The investigator should maintain all subject documents as specified in Essential Documents for conduct of a Clinical Subject (see ICH-GCP, section 8) and as required by the applicable regulatory requirement(s) after completion of the clinical subject so that they will be available for audits and inspections by the authorities. The investigator will be responsible for the storage.

The following retention periods will apply after completion or stop of the clinical subject:

- all essential documents and trial related data must be retained securely for at least 15 years,
- the subject identification list for at least 15 years,
- medical records and other source documents for the longest possible period allowed by the hospital, the institution or the private practice.

The investigator / institution should take arrangements to prevent accidental or premature destruction and illegitimate access to these documents.

To enable evaluations and / or audits from regulatory authorities or the sponsor, the investigator agrees to keep records, including the identity of all participating subjects (sufficient information to link records, e. g. CRFs and hospital records), all original signed informed consent forms, copies of all CRFs, serious adverse event forms, source documents, and detailed records of treatment disposition, drug accountability and adequate documentation of relevant correspondence (e. g. letters, meeting minutes, telephone calls reports).

The trial site will maintain a file of essential subject documentation (Trial site File). It is the responsibility of the site to retain copies of all completed CRFs for the subject and their trial file on site.

15 Reporting

15.1 Statistical Report

The statistical evaluation and the statistical report are performed, evaluated and signed by Prof. Dr. Hans-Helge Müller, Institut für medizinische Informationsverarbeitung, Biometrie und Epidemiologie. All data in this report are strictly confidential.

16 Definition of End of Trial

16.1 Regular End of the Trial

The regular end of the trial is defined as “Last Patient Last Visit“.

16.2 Termination of the Trial for Individual Subjects

If the clinical subject is prematurely terminated or suspended for any reason, the investigator should promptly inform the subjects and ensure appropriate therapy and follow-up for the subjects.
Where required by the applicable regulatory requirements, the competent authority(ies) and the ethics committee(s) will also be informed (this is usually done by the sponsor).

There are different grades of deviations from the study flow ranging from minor, major protocol violations, (such as delayed study visits, discontinuation of taking the study drug, taking medication listed under Prohibited therapy / concomitant medication) to complete withdrawal. Complete withdrawal should be a rare exception. In most situations the investigator will find a solution by allowing certain deviations from the protocol to meet the patient’s needs.

Whenever a subject is withdrawn from the trial, the circumstances of the withdrawal or discontinuation have to be recorded in detail in the CRF and a complete final examination as scheduled for the termination visit

should be conducted. The dosage of the IMP should be tapered off analogous to subjects completing the trial according to the protocol (see 10.2.3).

If a subject does not return for a scheduled visit, every effort should be made to contact the subject.

In any circumstance, every effort should be made to document subject outcome, if possible. The investigator should inquire about the reason for withdrawal. The subject has to be requested to return all unused investigational product(s), if applicable, and followed-up regarding any unresolved adverse events.

16.2.1 Termination by the Subject

Subjects may withdraw from the trial at any time at their own request without stating the reason(s) for withdrawal. They will experience no disadvantage as a result of this decision and no alternative therapy will be withheld by the investigator.

In this case the investigator is urged to ask the subject to return for an early termination visit and to document information as much as possible in the CRF.

16.2.2 Termination by the Investigator

- Subjects may also be withdrawn at any time at the discretion of the investigator for safety, behavioral, or administrative reasons at any time of the entire study period.
- Medically indicated e.g. because it is found that inclusion / exclusion criteria were violated;
- Continuation is unacceptable because risks outweigh the benefits;
- Pregnancy;
- Lack of compliance of the subject;
- Significant protocol violations;
- Logistical reasons (e.g. subject changes moves to a distant location).

16.3 Termination of One of the Treatment Arms or the Entire Trial

The sponsor / coordinating investigator is under obligation to monitor the progress of the clinical subject with regard to safety-relevant developments and, if necessary, initiate the premature termination of the clinical trial.

The sponsor / coordinating investigator will be supported in this responsibility by a Data and Safety Monitoring Board (DSMB).

A treatment arm or the entire clinical trial must be terminated prematurely if:

- New toxicological or pharmacological or SAEs invalidate the earlier benefit-to-risk ratio for the subject.
- Adverse events occurring in such severity and frequency that the proposed schedule can no longer be adhered to.
- Indications arise that the subjects' safety is no longer guaranteed.
- The question(s) addressed in the trial can be clearly answered on the basis of an interim analysis.

- An insufficient recruitment rate makes a successful conclusion of the clinical subject appear impossible.

The reasons for such a decision should be documented in written form.

16.4 Termination of the Trial in Individual Sites

Both the investigator and the sponsor delegated person have the right to terminate the trial at one of the centers at any time for instances:

- Unforeseeable circumstances have arisen at the trial site concerned that preclude the continuation of the clinical subject.
- The investigator considers that the resources for continuation are no longer available.
- The investigator considers that the continuation of the trial is no longer ethically or medically justifiable.
- Subject recruitment is inadequate.
- Serious problems arise with regard to the quality of the collected data which cannot be resolved.
- Withdrawal of the opinion of the EC and / or regulatory authority.

Premature termination at one of the trial centers does not automatically mean a termination of already enrolled trial subjects. A separate decision on further treatment must be made for each subject, depending on the overall situation. So, it has to be clarified that:

- An adequate further treatment and follow-up of already enrolled subjects must be ensured.
- The documentation of already enrolled subjects will be reviewed for completeness and plausibility. Queries may be raised for further clarification before the center is closed. These queries must be answered properly by the center.
- The competent authority(ies) and ethics committee(s) must be duly notified of the center's closure, including reasons, within the specified period(s).
- The trial center concerned will be closed in stages by the CRA when a decision has been made on the further treatment of the subjects concerned.

17 Monitoring, Audits and Inspections

During the clinical trial, quality control and quality assurance will be endured through monitoring, auditing and inspections by authorities.

17.1 Monitoring

To ensure accurate, complete, consistent, and reliable data, the investigator’s site(s) and trial procedures will be monitored by a representative of the sponsor. The sponsor’s representative will visit the site:

- to evaluate the progress and recruitment of the trial,
- to review the source documents and CRFs for protocol compliance, accuracy and validation,
- to assess facilities and equipment,
- to check for protocol compliance,
- to assure the AE/SAE reporting and
- to verify proper handling and dispensing of the IMP(s), and other factors.

The clinical monitor is also responsible for the transfer of the original CRFs to the sponsor. Frequency and scope of the monitoring visits will be defined in the Monitoring Plan for this trial which also includes the extent of source data verification that is required.

The investigator agrees to cooperate with the monitor to ensure that any problems detected in the course of these monitoring visits are addressed and resolved, and therefore ensures the accuracy and consistency of the trial with GCP and all applicable laws. The investigator allows the monitor to have access to all trial related original data and documents relevant for the monitoring of the trial.

17.2 Source Data Verification (SDV)

Source data verification will be performed in order to verify the accuracy and completeness of the entries on the case report form (CRF) by comparing them with the source data, and to ensure and increase the quality of the data. All data which are subject to SDV must have been entered in the medical record or, in the case of source documents, enclosed with the medical record. The investigators will afford the CRA access to the medical records for the performance of SDV.

Source data as defined by ICH-GCP include data such as hospital records, clinical and office charts, laboratory notes, memoranda, subjects' diaries or evaluation checklists, pharmacy dispensing records, recorded data from automated instruments, copies or transcriptions certified after verification as being accurate copies, microfiches, photographic negatives, microfilm or magnetic media, x-rays, and records kept at the pharmacy, at the laboratories and at medico-technical departments involved in the clinical subject.

For the following data of subject at minimum a 100% SDV has to be performed:

- Year of birth;
- Gender;
- (Serious) adverse events (AEs / SAEs);
- Main inclusion / exclusion criteria;
- Consent and
- Primary endpoints.

17.3 Audits and Inspections

In accordance with ICH GCP this trial may be selected for audit by representatives of the sponsor or for inspection by site responsible representatives of the local regulatory authority.

The investigator agrees to give the auditor access to all relevant documents for review and to support the sponsor to solve possible audit findings concerning the trial conduct at the respective site.

After every audit the auditee(s) will receive an audit confirmation by the auditor. This document has to be filed together with the trial documentation and has to be made available also to the authorities in case of an inspection.

At the end of the trial, a copy of the audit certificate(s) will be included in the final report.

18 Ethics and Good Clinical Practice

The trial will be conducted in accordance with the ICH Guideline for Good Clinical Practice, the relevant national regulations and the Declaration of Helsinki.

18.1 Responsibilities of the Sponsor

The sponsor will delegate the sponsors responsibilities and rights to a sponsor-delegated person (SDP). The SDP has to ensure that all legal requirements at each individual site will be fulfilled, before, during and after the end of the clinical trial. According to §§ 40 – 42 AMG the sponsor is responsible for obtaining the approval from the respective competent authority (BfArM) and the respective main research ethics committee ("federführende Ethikkommission") before initiation of the trial. In addition, the trial will be submitted to and approved by the appropriate independent research ethics committee for each participating center, prior to entering any patient into the trial.

According to § 4 Chap. 25 and § 40 Chap. 1 No. 5 AMG the sponsor announces a Coordinating Investigator ("Leiter der klinischen Prüfung"; LKP) who has more than two years of experience in the field of clinical trials and holds a medical license. Additionally, a principal investigator is announced for each trial site.

Before initiation of the trial (inclusion of the first patient), the trial has to be announced to the local regulatory authority(ies) according to § 67 AMG.

18.2 Responsibilities of the Investigator

By signing this protocol the local investigator declares his or her commitment:

- to not enrol any person dependent on him / her or the sponsor in accordance with the principles of ICH-GCP;
- to follow the regulations for data security according to § 7 Abs. 3, No. 15 GCP-V;
- to inform the subjects of the transmission of their pseudonymized data according to documentation and transmission obligations (§ 12 and § 13 GCP-V) and to make sure that subjects unwilling to give consent to the processing of their data are not included into the trial;
- to certify that he / she was informed of the pharmacological-toxicological issues and risks of the clinical subject according to § 40 Abs. 1, Satz 3 No. 7 AMG;
- to be qualified by education, training and experience to assume responsibility for the proper conduct of the subject;
- to be thoroughly familiar with the appropriate use of the trial drug(s), as described in the protocol, the product information and other information sources provided by the sponsor;
- to be aware of and comply with GCP and the applicable regulatory requirements and
- to maintain a list of appropriately qualified persons to whom the investigator has delegated significant subject related duties (if applicable).

18.3 Ethics Committee and Competent Authority(ies)

The clinical trial protocol and amendments have to be approved by the Competent Authorities (CA), in addition to protocol amendments to the subject information. Informed consent and any other written information to be provided to the trial patients have to be approved by the respective main research ethics committee ("federführende Ethikkommission") and by the appropriate independent research ethics committee for each participating site.

The sponsor-delegated person will authorize the CSC to submit the documents to the Ethics Commitee(s) (EC) and to the CA.

A copy of the written approval must be received by the sponsor before recruitment of subjects into the trial and shipment of the trial drug.

Any substantial amendments to the protocol or subsequent changes to the informed consent form as a result of changes to the protocol must also be sent to the EC / CA. Records of the EC review and opinion of all documents pertaining to this trial must be kept on file by the investigator and are subject to regulatory authority and / or sponsor inspection during or after completion of the trial.

The sponsor-delegated person will provide a safety update of the trial to the EC(s) / CA, including line listing, individual reports of SUSARs, if applicable, annually or more frequently if requested.

18.4 Compliance with the Protocol

The investigator should conduct the clinical trial in compliance with this protocol. For this purpose, the document will be signed by the sponsor and the investigator. As a general rule, the investigator should not deviate from the protocol or make amendments to the protocol without the agreement of the sponsor / authority / ethics committee (unless subject safety is at risk, see below).

Any deviations from the approved protocol should be documented and explained by the investigator or an individual who is designated by the investigator.

The investigator may deviate from the protocol or make an amendment to the protocol without prior approval of the ethics committee to eliminate immediate risks to the subjects. The deviation or amendment should subsequently be reported to the ethics committee, the sponsor or sponsor delegated person and, if necessary, the competent authority, giving reasons.

18.5 Notification of General Amendments to the Protocol

The sponsor can make general amendments to the protocol after the clinical trial has started. These may be of an administrative nature (logistical / administrative amendments) or substantial.

Substantial Amendments are changes that likely affect and / or change:

- the safety of the persons concerned,
- the interpretation of the scientific trial documents or the scientific informational value of the trial results,
- the nature of management or conduct of the clinical subject (e.g. change of coordinating investigator (German LKP), sponsor or sponsors deputy),
- the pharmaceutical quality or safety of the investigational medicinal products,
- the risk assessments concerning the health of persons who are not concerned, or the environment, in clinical subjects with drugs consisting of or containing genetically modified organisms.

Substantial Amendments require a new authorization of the Competent Authority and a new favourable opinion by the Ethics Committee.

The clinical trial may only be continued when a favourable opinion has been obtained from the competent ethics committee and if the competent authority has not raised any objections accompanied by reasons.

If applicable, an updated Informed Consent Form has to be signed by all subjects enrolled in the trial who are affected by the amendment.

Amendments which only have to be approved by the EC (e.g. changes in an advertisement for subjects to participate in the trial or changes in facilities for the trial, also will be notified to the CA with the comment “For

information only". Similarly, the EC will be informed of any substantial amendments for which only the CA is responsible (e.g. quality data).

If administrative protocol changes (e.g. change of monitoring, telephone numbers) are necessary, the EC and CA will be notified only.

18.6 Notification of the end of the trial

The end of the clinical trial is the date of the last visit of the last subject undergoing the trial.

At the end of the trial, the CSC will notify the EC(s) / CA about the trial completion. A copy of all reports submitted to the EC will be sent to the sponsor. Within one year of the end of the complete trial a summary of the trial report will be provided to the CA(s) and EC(s).

18.7 Annual Safety Report

Together with the notification of the end of the trial and once a year the sponsor delegated person will provide the CA(s) and EC(s) in the country concerned with a listing of all suspected serious adverse reactions (SAEs) which occurred over the trial period of the subject's safety.

18.8 Subject Information and Informed Consent

According to § 40 of the German "Arzneimittelgesetz (AMG)" every participating patient will be informed of the nature, importance, risks and consequences of the trial by the local investigator. The local investigator is responsible for obtaining written informed consent prior to randomization into the trial. Patients unable to give informed consent will not be included.

Patients must understand that it is their own free will to participate and that they can withdraw consent at any time without giving reasons. Patients must also understand that they will experience no disadvantage as a result of this decision and that no alternative therapy will be withheld by the investigator.

On the other hand, by signing the consent form patients give their consent to the evaluation and usage of their personal data according to § 40 Abs. 2a AMG.

The personally signed and dated informed consent forms will be filed in the Investigator Site File at each site. A copy of the informed consent form will be given to the patient and a copy will be held in the patient's medical record. The existence of a written informed consent will have to be confirmed at the time of randomization.

Should any new data provide information on the safety profile of any of the trial medication leading to significant changes in the risk/benefit ratio, the patient information sheet and informed consent form will be checked and adjusted.

18.9 Subject Insurance

Every subject participating in the trial is insured according to § 40 Abs. 1 Nr. 8 and Abs. 3 AMG against injuries to health which may occur during the trial.

Excluded from this, however, are injuries to health and deterioration of illnesses already in existence which would have continued to exist even if the subject had not taken part in the clinical trial.

Insurance coverage is jeopardized if the subject fails to immediately report to the investigator or responsible physician any injury to health which might have resulted from the participation in the clinical trial, or if she or he undergoes any other medical treatment (except for emergency treatment) without the investigator's knowledge before her or his participation in the clinical trial has officially ended. In case of an emergency treatment the subject is obliged to inform the investigator as soon as possible after the treatment.

Any injury to health which might have occurred as a result of participating in the clinical trial must be reported by the subject to the investigator without delay. In all cases the investigator is obliged to make a report to the sponsor, who will then inform the insurer.

The subject's insurance will be arranged by the sponsor-delegated person. The insurer will be:

Name of Insurer:	HDI Gerling Industrie Versicherung AG
Insurance Number:	39 130537 03026
Address:	Niederlassung Düsseldorf
	Am Schönenkamp 45
	D-40599 Düsseldorf

This insurance covers trial-related injuries to health up to a maximum of 500,000 Euro per subject.

18.10 Data Protection and Subject Confidentality

The collection, transmission, archiving and evaluation of personal data in this clinical trial are done according to locally applicable laws (Data Protection Act). Prior to trial participation each subject must be informed by the investigator about the trial and must give his or her written informed consent.

The subjects must be informed of the following:

- Any patient-related data in this trial are handled confidentially (pseudonymized) and will only be transmitted to the coordinating investigator / sponsor / sponsor-delegated person / data monitoring safety board for scientific and adverse event evaluation and
- the responsible regulatory authority(ies) (local authority(ies) / BfArM or PEI), the ECs of the trial sites and the European Data Base (EudraCT data base) for verifying the proper conduct of the trial and for assessment of trial results and adverse events.
- During monitoring, audits or inspections representatives of the sponsor (monitor, auditor) or of the local regulatory authority(ies) must have direct access to personal data. In this case, the investigator is released from confidential medical communication.

18.11 Financing of the Trial

The present trial is an investigator-initiated trial (IIT). The trial is financially sponsored by the Federal Ministry of Education and Research ("Bundesministerium für Bildung und Forschung, BMBF").

18.11.1 Trial Agreement / Investigator Compensation

According to ICH-GCP 4.9.6, a trial agreement on the conduct of the clinical subject and the compensation for conducting the subject will be signed between the sponsor (donor) of the clinical subject and the investigators including their heads of administration (donee). A compensation will be paid for each fully documented, completed case.

18.11.2 Reimbursement of Subjects

Subjects will be compensated for their travel expenses in the context of trial site visits.

19 Trial Reports

After completion of the analysis by the responsible biostatistician, the final integrated medical and statistical report will be prepared and signed jointly with the biostatistician.

Except when required by law, no one will disclose a result of the clinical subject to third parties unless all parties involved have first agreed on the results of the analysis and their interpretation.

The final trial report will be written and signed in co-operation between the sponsor / coordinating investigator and the responsible statistician. All data in this report are strictly confidential.

20 Publication

20.1 Publication Policy

Please note that publication of the trial results is in accordance with the Clinical Trial Agreement between trial site and sponsor.

The results of the trial shall be published. The publication or a lecture based on the data needs a previous annotation and approval of the Coordinating Investigator. All patient-related data need to be published in a pseudonymous form.

21 References

Best, C, Eckhardt-Henn, A, Diener, G, Bense, S, et al. 2006, “Interaction of somatoform and vestibular disorders.” *Journal of Neurology, Neurosurgery, and Psychiatry*, vol. 77, no. 5, pp. 658-664.

Bisdorff, AR 2004, “Treatment of migraine related vertigo with lamotrigine an observational study.” *Bulletin De La Société Des Sciences Médicales Du Grand-Duché De Luxembourg*, no. 2, pp. 103-108.

Brandes, JL, Saper, JR, Diamond, M, Couch, JR, et al. 2004, “Topiramate for migraine prevention: a randomized controlled trial.” *JAMA: The Journal of the American Medical Association*, vol. 291, no. 8, pp. 965-973.

Carmona, S & Settecase, N 2005, “Use of topiramate (topamax) in a subgroup of migraine-vertigo patients with auditory symptoms.” *Annals of the New York Academy of Sciences*, vol. 1039, pp. 517-520.

Diener, H-C, Katsarava, Z & Limmroth, V 2008, “[Current diagnosis and treatment of migraine].” *Der Ophthalmologe: Zeitschrift Der Deutschen Ophthalmologischen Gesellschaft*, vol. 105, no. 5, pp. 501-508; quiz 509-510.

Diener, H-C & Putzki, N (eds.) 2008, *Leitlinien für Diagnostik und Therapie in der Neurologie* 4th ed., Retrieved December 16, 2011, from http://www.dgn.org/component/content/article/18-leitlinien/461-leitlinien-der-dgn-therapie-der-migraene.html?q=migr%C3%A4ne

Dieterich, M & Brandt, T 1999, “Episodic vertigo related to migraine (90 cases): vestibular migraine?” *Journal of Neurology*, vol. 246, no. 10, pp. 883-892.

Evers, S, Afra, J, Frese, A, Goadsby, PJ, et al. 2009, “EFNS guideline on the drug treatment of migraine--revised report of an EFNS task force.” *European Journal of Neurology: The Official Journal of the European Federation of Neurological Societies*, vol. 16, no. 9, pp. 968-981.

Evers, S 2008, “Treatment of migraine with prophylactic drugs.” *Expert Opinion on Pharmacotherapy*, vol. 9, no. 15, pp. 2565-2573.

Fairclough, DL 2002, *Design and analysis of quality of life studies in clinical trials*, CRC Press, Boca Raton, Florida, USA.

Furman, JM, Marcus, DA & Balaban, CD 2003, “Migrainous vertigo: development of a pathogenetic model and structured diagnostic interview.” *Current Opinion in Neurology*, vol. 16, no. 1, pp. 5-13.

Johnson, GD 1998, “Medical management of migraine-related dizziness and vertigo.” *The Laryngoscope*, vol. 108, no. 1 Pt 2, pp. 1-28.

Lempert, T & Neuhauser, H 2005, “Migrainous vertigo.” *Neurologic Clinics*, vol. 23, no. 3, pp. 715-730, vi.

Marcus, DA, Kapelewski, C, Rudy, TE, Jacob, RG & Furman, JM 2004, “Diagnosis of migrainous vertigo: validity of a structured interview.” *Medical Science Monitor: International Medical Journal of Experimental and Clinical Research*, vol. 10, no. 5, pp. CR197-201.

Müller, H-H & Schäfer, H 2004, “A general statistical principle for changing a design any time during the course of a trial.” *Statistics in Medicine*, vol. 23, no. 16, pp. 2497-2508.

Müller, H-H & Schäfer, H 2001, “Adaptive group sequential designs for clinical trials: combining the advantages of adaptive and of classical group sequential approaches.” *Biometrics*, vol. 57, no. 3, pp. 886-891.

Neuhauser, HK, Radtke, A, von Brevern, M, Feldmann, M, et al. 2006, “Migrainous vertigo: prevalence and impact on quality of life.” *Neurology*, vol. 67, no. 6, pp. 1028-1033.

Neuhauser, H, Leopold, M, von Brevern, M, Arnold, G & Lempert, T 2001, “The interrelations of migraine, vertigo, and migrainous vertigo.” *Neurology*, vol. 56, no. 4, pp. 436-441.

Neuhauser, H, Radtke, A, von Brevern, M & Lempert, T 2003, “Zolmitriptan for treatment of migrainous vertigo: a pilot randomized placebo-controlled trial.” *Neurology*, vol. 60, no. 5, pp. 882-883.

Neuhauser, HK 2007, “Epidemiology of vertigo.” *Current Opinion in Neurology*, vol. 20, no. 1, pp. 40-46.

Olesen, J 2005, “Vertigo and dizziness related to migraine: a diagnostic challenge.” *Cephalalgia: An International Journal of Headache*, vol. 25, no. 9, pp. 761-762; author reply 762-763.

Reploeg, MD & Goebel, JA 2002, “Migraine-associated dizziness: patient characteristics and management options.” *Otology & Neurotology: Official Publication of the American Otological Society, American Neurotology Society [and] European Academy of Otology and Neurotology*, vol. 23, no. 3, pp. 364-371.

Strupp, M & Brandt, T 2008, “Diagnosis and treatment of vertigo and dizziness.” *Deutsches Ärzteblatt International*, vol. 105, no. 10, pp. 173-180.

Strupp, M, Hupert, D, Frenzel, C, Wagner, J, et al. 2008, “Long-term prophylactic treatment of attacks of vertigo in Menière’s disease--comparison of a high with a low dosage of betahistine in an open trial.” *Acta Oto-Laryngologica*, vol. 128, no. 5, pp. 520-524.

Strupp, M, Versino, M & Brandt, T 2010, “Vestibular migraine.” *Handbook of Clinical Neurology / Edited by P.J. Vinken and G.W. Bruyn*, vol. 97, pp. 755-771.

Warninghoff, JC, Bayer, O, Ferrari, U & Straube, A 2009, “Co-morbidities of vertiginous diseases.” *BMC Neurology*, vol. 9, p. 29.

Wieser, T, Wolff, R, Hoffmann, KP, Schulte-Mattler, W & Zierz, S 2004, “Persistent ocular motor disturbances in migraine without aura.” *Neurological Sciences: Official Journal of the Italian Neurological Society and of the Italian Society of Clinical Neurophysiology*, vol. 25, no. 1, pp. 8-12.

Wörz, R, Reinhardt-Benmalek, B, Föh, M, Grotemeyer, KH & Scharafinski, HW 1992, “[Prevention of migraine using bisoprolol. Results of a double-blind study versus metoprolol].” *Fortschritte Der Medizin*, vol. 110, no. 14, pp. 268-272.

22 Questionnaire: Dizziness Handicap Inventory (DHI)

Beeinträchtigung durch Schwindel

Dieser Fragebogen dient dazu, die Probleme herauszufinden, die Sie wegen Ihres Schwindels oder Ihrer Gleichgewichtsprobleme haben können. Beantworten Sie bitte jede Frage entweder mit ja, nein oder manchmal. Beantworten Sie jede Frage nur in Bezug auf Ihr Schwindel- oder Gleichgewichtsproblem.

	Ja	Manch-mal	Nein
Verstärken sich Ihre Probleme, wenn Sie nach oben schauen?	☐	☐	☐
Fühlen Sie sich wegen Ihrer Probleme frustriert?	☐	☐	☐
Schränken Sie wegen Ihrer Probleme geschäftliche oder private Reisen ein?	☐	☐	☐
Verstärken sich Ihre Probleme, wenn Sie einen Gang im Supermarkt entlang gehen?	☐	☐	☐
Haben Sie wegen Ihrer Probleme Schwierigkeiten beim ins Bett gehen oder beim Aufstehen aus dem Bett?	☐	☐	☐
Schränken Ihre Probleme Sie deutlich ein, an gesellschaftlichen Aktivitäten teilzunehmen (z.B. auswärts essen gehen, Einladungen folgen, zu Parties gehen, ins Kino gehen, Theater oder Konzerte besuchen)?	☐	☐	☐
Haben Sie wegen Ihrer Probleme Schwierigkeiten beim Lesen?	☐	☐	☐
Verstärken sich Ihre Probleme bei anspruchsvolleren Aktivitäten z.B. im Sport, beim Tanzen oder bei Hausarbeiten?	☐	☐	☐
Haben Sie wegen Ihrer Probleme Angst, das Haus ohne Begleitung zu verlassen?	☐	☐	☐
Sind Sie wegen Ihrer Probleme schon einmal in eine peinliche Situation geraten?	☐	☐	☐
Verstärken schnelle Kopfbewegungen Ihre Probleme?	☐	☐	☐

Meiden Sie die Höhe wegen Ihrer Probleme (zum Beispiel: Berge, Hochhaus, Leiter, Gerüst)?	☐	☐	☐
Verstärken sich Ihre Probleme, wenn Sie sich im Bett drehen?	☐	☐	☐
Haben Sie wegen Ihrer Probleme Schwierigkeiten, anstrengende Haus- oder Gartenarbeit zu erledigen?	☐	☐	☐
Befürchten Sie, dass andere Leute wegen Ihrer Probleme denken, Sie seien betrunken?	☐	☐	☐
Haben Sie wegen Ihrer Probleme Schwierigkeiten, alleine spazieren zu gehen?	☐	☐	☐
Verstärken sich Ihre Probleme, wenn Sie auf einem Trottoir/Bürgersteig gehen?	☐	☐	☐
Ist es wegen Ihrer Probleme schwierig für Sie, sich zu konzentrieren?	☐	☐	☐
Ist es wegen Ihrer Probleme für Sie schwierig, sich im Dunkeln in Ihrer Wohnung zu bewegen?	☐	☐	☐
Haben Sie wegen Ihrer Probleme Angst, alleine zu Hause zu bleiben?	☐	☐	☐
Fühlen Sie sich wegen Ihrer Probleme behindert/eingeschränkt?	☐	☐	☐
Belasten Ihre Probleme die Beziehung zu Familienmitgliedern oder Freunden?	☐	☐	☐
Fühlen Sie sich auf Grund Ihrer Probleme deprimiert?	☐	☐	☐
Werden Sie durch Ihre Probleme beeinträchtigt, Ihre Aufgaben im Beruf oder Haushalt wahrzunehmen?	☐	☐	☐
Verstärken sich Ihre Probleme, wenn Sie sich nach vorne beugen?	☐	☐	☐
