# Supplementary material for: Results and lessons learnt from a randomized controlled trial: prophylactic treatment of vestibular migraine with metoprolol (PROVEMIG)
Source: Trials. 2019 Dec 30;20:813. doi: 10.1186/s13063-019-3903-5 (PMC6937687; doi:10.1186/s13063-019-3903-5)
Supplement: Supplementary file 3 — Additional file 3. Patient symptom diary (original German version; word-for-word English translation). [file 13063_2019_3903_MOESM3_ESM.pdf]

English translation

LMU | KLINIKUM DER UNIVERSITÄT MÜNCHEN | CAMPUS GROSSHADERN GERMAN CENTER FOR VERTIGO AND BALANCE DISORDERS | IFB LMU GEFÖRDERT VOM Bundesministerium für Bildung und Forschung

Dizziness diary

PROVEMIG-Trial Patient-ID: ______________________ Month/Year: ___/______

**Date**	**Time of day**	**Duration of the Attack** in hours, minutes	**Type and severity of vertigo**<br>S postural vertigo (like on a ship)<br>D rotatory vertigo (like in a carousel)<br>G gait ataxia<br>B lightheadedness<br>×mild, ××moderate, ×××severe	**Accompanying symptoms**, e. g.<br>A headache<br>B nausea<br>C vomiting<br>D photo- or phonophobia<br>E diplopia<br>F other visual symptoms<br>G tendency to fall<br>H other: __________________<br>**Remarks**<br>action taken<br>medication
1				
2				
3				
4				
5				
6				
7				
8				
9				
10				
11				
12				
13				
14				
15				
16				
17				
18				
19				
20				
21				
22				
23				
24				
25				
26				
27				
28				
29				
30				
31				

Instructions for using the dizziness diary

General

The dizziness diary is critical to assessing your treatment success. Every time you visit the study center, the investigator will go over it with you to interpret its content correctly. Please also have it ready for the phone interviews. At the time of issue, the investigator will enter your patient identification number at the top, which has been assigned to you for the PROVEMIG study, and which you will also find on your study medication. Please also make sure that month and year (e.g. Jan - 2012) are not missing.

Structure

For each calendar month a diary sheet is kept. In the left column you will find the date (day). If an attack occurs, enter the time, duration, nature and accompanying complaints in this line in the boxes provided. To save space, abbreviations for dizziness and concomitant symptoms are already given in the header. If your attacks are regularly accompanied by symptoms that are not listed there, you can assign more abbreviations (see H others: _________). Specify the severity behind the type of dizziness using x to xxx (mild to severe).

Undocumented periods (e.g. because they have been missed or not covered by the diary sheet) have to be crossed out.

The following example shows entries of a patient who

- came on the 3rd for a visit. The days before that are cancelled, as they were before the beginning of study, or were covered by a diary sheet, which was already delivered at the last visit.
- was symptom-free on the 3rd (day of the visit).
- suffered a 30-minute rotatory vertigo, headache and nausea on the 4th, and took Ibuprofen.
- was mildly lightheaded on the 5th since awakening at 8 a.m. to noon.

The duration refers to the duration of the dizziness given in the 4th column. If the duration of concomitant symptoms deviates significantly, please note this in the right column (e.g. headache 4 h).

**Date**	**Time of day**	**Duration of the Attack** in hours, minutes	**Type and severity of vertigo**<br>S postural vertigo (like on a ship)<br>D rotatory vertigo (like in a carousel)<br>G gait ataxia<br>B lightheadedness<br>×mild, ××moderate, ×××severe	**Accompanying symptoms,** e. g.<br>A headache<br>B nausea<br>C vomiting<br>D photo- or phonophobia<br>E diplopia<br>F other visual symptoms<br>G tendency to fall<br>H other: ___________________ **Remarks**<br>action taken<br>medication
~~1~~				
~~2~~				
3				
4	16:30	30 min	D xx	A, B, Ibuprofen 400 mg eingenommen
5	08:00	4 h	B x	
…				
31				

If you have any questions, please contact your investigator.

English translation of Version 1.0 from 18.01.2012.

Original version

LMU | KLINIKUM DER UNIVERSITÄT MÜNCHEN | INTEGRIERTES FORSCHUNGS- UND BEHANDLUNGSZENTRUM FÜR SCHWINDEL, GLEICHGEWICHTS- UND AUGENBEWEGUNGSSTÖRUNGEN | IFB LMU GEFÖRDERT VOM Bundesministerium für Bildung und Forschung

Schwindeltagebuch

PROVEMIG-Studie Patienten-ID: ______________________ Monat/Jahr: ___/______

**Datum**	**Uhrzeit**	**Dauer der Attacke** in Stunden, Minuten	**Art und Stärke des Schwindels**<br>S Schwankschwindel (wie auf einem Schiff)<br>D Drehschwindel (wie in einem Karusell)<br>G Gangunsicherheit<br>B Benommenheitsgefühl<br>x leicht, xx mittel, xxx stark	**Begleitende Beschwerden**, z. B.<br>A Kopfschmerz<br>B Übelkeit<br>C Erbrechen<br>D Licht- oder Lärmempfindlichkeit<br>E Doppelbilder<br>F andere Sehstörungen<br>G Sturzneigung<br>H andere: ________________<br><br>**Bemerkungen**<br>Maßnahmen, Medikamente
1				
2				
3				
4				
5				
6				
7				
8				
9				
10				
11				
12				
13				
14				
15				
16				
17				
18				
19				
20				
21				
22				
23				
24				
25				
26				
27				
28				
29				
30				
31				

Hinweise zum Schwindeltagebuch

Allgemeines

Das Schwindeltagebuch ist zur Beurteilung Ihres Behandlungserfolgs von entscheidender Bedeutung. Bei jedem Besuch im Studienzentrum wird der Prüfarzt es mit Ihnen durchgehen, um den Inhalt richtig interpretieren zu können. Bitte halten Sie es auch für die Telefoninterviews bereit. Bei Ausgabe trägt der Prüfarzt oben Ihre Patienten-Identifikations-nummer ein, die Ihnen für die PROVEMIG-Studie zugewiesen wurde und die Sie auch auf Ihrer Studienmedikation wiederfinden. Bitte achten Sie auch darauf, daß Monat und Jahr (z. B. Jan – 2012) nicht fehlen.

Aufbau

Für jeden Kalendermonat wird ein Tagebuchblatt geführt. In der linken Spalte finden Sie das Datum (Tag). Wenn eine Attacke auftritt, tragen Sie Uhrzeit, Dauer, Art und begleitende Beschwerden in dieser Zeile in die vorgesehenen Kästchen ein. Um Platz zu sparen, sind in der Kopfzeile bereits Abkürzungen für Schwindelarten und Begleitsymptome vorgegeben. Falls mit Ihren Attacken regelmäßig nicht dort gelistete Begleitsymptome auftreten, können Sie weitere Abkürzungen vergeben (siehe H andere: ______). Die Schwere geben Sie hinter der Schwindelart mit x bis xxx (leicht bis stark) an.
Zeiträume, die nicht dokumentiert wurden (z. B. weil sie versäumt wurden oder die durch das Tagebuchblatt nicht abgedeckt wurden), sind zu streichen.

Das folgende Beispiel zeigt Einträge eines Patienten, der

- am 3. zur Visite kam. Die Tage davor sind gestrichen, da sie vor Studienbeginn lagen, oder von einem Tagebuchblatt abgedeckt wurden, das bei der letzten Visite schon abgegeben wurde.
- am 3. (Tag der Visite) beschwerdefrei war
- am 4. eine 30minütige Drehschwindelattacke, Kopfschmerzen und Übelkeit erlitten und Ibuprofen eingenommen hat
- am 5. seit dem Aufwachen um 8 Uhr bis mittags leicht benommen war

Die Dauer bezieht sich auf die Dauer des in der 4. Spalte angegebenen Schwindels. Sollte die Dauer von Begleitsymptomen davon deutlich abweichen, notieren Sie dies bitte in der rechten Spalte (z. B. Kopfschmerz 4 h).

**Datum**	**Uhrzeit**	**Dauer der Attacke** in Stunden, Minuten	**Art und Stärke des Schwindels**<br>S Schwankschwindel (wie auf einem Schiff)<br>D Drehschwindel (wie in einem Karusell)<br>G Gangunsicherheit<br>B Benommenheitsgefühl<br>x leicht, xx mittel, xxx stark	**Begleitende Beschwerden**, z. B.<br>A Kopfschmerz<br>B Übelkeit<br>C Erbrechen<br>D Licht- oder Lärmempfindlichkeit<br>E Doppelbilder<br>F andere Sehstörungen<br>G Sturzneigung<br>H andere: ________________	**Bemerkungen**<br>Maßnahmen, Medikamente
1					
2					
3					
4	16:30	30 min	D xx	A, B, Ibuprofen 400 mg eingenommen	
5	08:00	4 h	B x		
...					
31					

Bei Unklarheiten wenden Sie sich bitte an Ihren Prüfarzt.

Version 1.0 vom 18.01.2012
