# Supplementary material for: Results and lessons learnt from a randomized controlled trial: prophylactic treatment of vestibular migraine with metoprolol (PROVEMIG)
Source: Trials. 2019 Dec 30;20:813. doi: 10.1186/s13063-019-3903-5 (PMC6937687; doi:10.1186/s13063-019-3903-5)
Supplement: Supplementary file 4 — Additional file 4. Procedural and statistical methods. [file 13063_2019_3903_MOESM4_ESM.docx]

**SUPPLEMENTARY MATERIALS**

**Results and lessons learnt from a randomized controlled trial:**

**Prophylactic treatment of vestibular migraine with metoprolol (PROVEMIG)**

Otmar Bayer *****, Christine Adrion *****, Amani Al Tawil, Ulrich Mansmann, Michael Strupp,
on behalf of the *PROVEMIG* investigators

***** Contributed equally and share first authorship

**The PDF file includes:**

[S1. Investigators and Participating Centres 2](#_Toc3218495)

[S2. Procedural and Statistical Methods 3](#_Toc3218496)

[2.1 Figure S1. Cumulative patient enrolment since the start of the trial 3](#_Toc3218497)

[2.2 Figure S2. Missingness Map (vertigo attacks) 3](#_Toc3218498)

[2.3 Determination of the fixed sample size – further details 5](#_Toc3218499)

[S3. Secondary Efficacy Outcomes 6](#_Toc3218500)

[3.1 Subjective visual vertical (SVV); Pursuit eye movement test 6](#_Toc3218501)

[3.2 Quality of Life (QoL): Dizziness Handicap Inventory (DHI) questionnaire − Definition of the Total Score 6](#_Toc3218502)

[References 7](#_Toc3218503)

# Investigators and Participating Centres

**Study centre(s):** Before trial commencement, 8 centres in Germany gave consent to participate in the recruitment process. In the end, 6 centres were initiated and screened for eligible patients (LMU Munich, Celle, Altötting-Burghausen, Parkklinik Berlin-Weißensee, Tübingen, Essen), 4 of them (LMU Munich, Celle, Altötting-Burghausen, Essen) allocated patients to the trial.

*The following principal investigators participated in the PROVEMIG trial:*

Prof. Dr. med. Dr. h.c. Michael Strupp – Hospital of the University of Munich, Clinic for Neurology and German Center for Vertigo and Balance Disorders, 81377 Munich, Germany

Prof. Dr. med. Hans-Christoph Diener – Hospital of the University of Essen, Department of Neurology, 45130 Essen, Germany

Prof. Dr. med. Hubert Löwenheim – Hospital of the University of Tübingen, Department of ENT, 72076 Tübingen, Germany

Prof. Dr. med. Thomas Lempert – Schlosspark-Klinik, Department of Neurology, 14059 Berlin, Germany

Prof. Dr. med. Wolfgang Heide – Allgemeines Krankenhaus, Neurology, 29223 Celle, Germany

Prof. Dr. med. Holger Rambold – Kreiskliniken Altötting-Burghausen, Neurologische Klinik, 84503 Altötting, Germany

# Procedural and Statistical Methods

## Figure S1. Cumulative patient enrolment since the start of the trial

Figure S1. Cumulative patient enrolment since the start of the trial. The solid back line depicts the overall cumulative frequency (total number of subjects randomized over time, aggregated over all study sites). The dashed lines (red, blue, light blue, grey colour) represent recruitment of the 4 recruiting sites. The black dotted vertical line represents the target end of June 2017 (stop of recruitment). First patient first visit was on 20 Jun 2012. Last patient first visit was on 10 April 2017.

## Figure S2. Missingness Map (vertigo attacks)

The proportion of missings over time (i.e. per 30-day interval 1, 2, …, 6) can be visualized by means of a missingness map: This plot displays the missingness patterns concerning diary information across the 6-month time period. In the case of missing data for at least one 30-day interval, corresponding gaps are indicated in dark grey; time periods with available data are displayed in light grey.

The proportion of intermittent missings was rather low for each treatment arm. The proportion of monotone missing diary information was higher in the placebo group. In total, 13 patients did not provide any post-randomization data (9 patients on placebo; 4 patients on metoprolol). The percentage of missings concerning vertigo attacks over the whole 6-month treatment period was higher in the placebo compared to the metoprolol group.
Altogether, the proportion of monotone (and intermittent) missings was not higher than expected for symptomatic trials assessing the ability of an intervention to provide symptom relief from the condition.

Figure S2. Missingness Maps showing where missingness occurs in the vertigo attack dataset (ITT sample) within the time period of primary interest (interval 4, 5, 6).
The figures display monotone and intermittent missing data patterns concerning diary information. Light grey: diary data available; dark grey: missing data. *x*-axis: 30-day time periods *t* = 1, 2, …, 6. *y*-axis: patients are sorted according to their original PatID within each treatment group.

## Determination of the fixed sample size – further details

A fixed sample size calculation was performed for the primary efficacy outcome number of vertigo attacks. Three parameters influence the sample size of the study which uses the Wilcoxon (Mann-Whitney) rank-sum test for the statistical decision: the level of significance, the power of the two-sided test, and the probability that an observation X_M_ in the metoprolol group is less than an observation X_P_ in the placebo group. Here, the observations are the values of the primary endpoint expressed in attacks per months based on a 3-month assessment period at the end of the 6-month treatment period. For the primary endpoint a reduction of 1 attack per month due to metoprolol was regarded as the minimal clinically important difference (MCID) and set the target difference.[1, 2] However, the clinical relevance of reductions less than 1 attack per month is debatable. Brandes and colleagues[3] reported reductions of a little more than 1 attack per month by medication over up to 18 weeks (more than 40% drop off before 18 weeks medication) in migraine prevention where the baseline attack frequency was restricted to 3 to 12 attacks per months. They observed a mean of around 4 attacks per months during the medication period with a standard deviation of around 3 attacks per months.
In patients suffering from vestibular migraine, the number of vertigo attacks per month rarely exceeds 10. Restricting recruitment to patients with vestibular migraine with a history of vertigo attacks of 2 to 10 attacks per months, we expected a standard deviation of the first primary endpoint corresponding to a value of 2 to 2.5 vertigo attacks per month.
A sample size of **106 patients in each group** will have 80% power to detect a probability of 0.389 that an observation X_M_ is less than an observation X_P_ using a Mann-Whitney test with a 5% two-sided significance level. The probability of *P*(X_M_ < X_P_)=0.611 was calculated with a presumed normal distribution and difference in means of 1 and a standard deviation of 2.5 (nQuery Advisor 7.0). On the basis of our experience with patient compliance in previous studies and routine treatment, we assumed a drop-out rate of about 20%. Thus, the fixed target sample size was **a total of 266 patients** (133 in each treatment group) to be allocated.

# Secondary Efficacy Outcomes

## Subjective visual vertical (SVV); Pursuit eye movement test

The SVV is a method to evaluate a patient's capacity to determine if an object is aligned vertically, without any visual vertical reference relying on its graviceptive pathways. Patients were asked to align a line vertically, and the deviation from the objective vertical axis was measured in degrees (normal range (mean ± 2 SDs) of the SVV is 0° ± 2.5°). The pursuit eye movement test aims to differentiate the movement of the eye whether being saccadic (i.e. pathologic state) or smooth.

## Quality of Life (QoL): Dizziness Handicap Inventory (DHI) questionnaire − Definition of the Total Score

To assess the impact of impairment the patients were asked to fill out the 25-item DHI questionnaire.

The original DHI total score (range: 0 to 100 points) consists of three subscales: *functional subscale (F), emotional subscale (E) and a physical subscale (P)*. The top score is 100 (maximum perceived disability), the bottom score is 0 (no perceived disability).

The subjective measure of the patient’s perception of handicap due to the dizziness can be categorized as follows [4]:

- 16−34 points (mild handicap)
- 36−52 points (moderate handicap)
- 54+ points (severe handicap).

For reach of the 25 items, a “yes/always” response is scored 4 points, a “sometimes” response 2 points, and a “no” response 0 points.

To deal with missing items, we used the derived ***DHI mean total score*** (DHI Total_mean_) as outcome variable averaging for the number of answered questions:

DHI Total_mean_ = $(\frac{1}{\sum_{i} {item}_{i}\neq NA})\sum_{i=1}^{25} {item}_{i}$

where *NA* denotes a missing answer. In R code [5] this means: mean(., na.rm = T).

# References

1. Cook JA, Hislop J, Altman DG, Fayers P, Briggs AH, Ramsay CR, Norrie JD, Harvey IM, Buckley B, Fergusson D *et al*: **Specifying the target difference in the primary outcome for a randomised controlled trial: guidance for researchers**. *Trials* 2015, **16**(1):12.

2. Cook JA, Julious SA, Sones W, Hampson LV, Hewitt C, Berlin JA, Ashby D, Emsley R, Fergusson DA, Walters SJ *et al*: **DELTA2 guidance on choosing the target difference and undertaking and reporting the sample size calculation for a randomised controlled trial**. *BMJ* 2018, **363**.

3. Brandes JL, Saper JR, Diamond M, Couch JR, Lewis DW, Schmitt J, Neto W, Schwabe S, Jacobs D: **Topiramate for migraine prevention: a randomized controlled trial**. *JAMA* 2004, **291**(8):965-973.

4. Jacobson GP, Newman CW: **The development of the Dizziness Handicap Inventory**. *Arch Otolaryngol Head Neck Surg* 1990, **116**(4):424-427.

5. R Development Core Team: ***R*: A language and environment for statistical computing**. In*.* Vienna, Austria: R Foundation for Statistical Computing, <http://www.R-project.org/>; 2018.
